# Dynamic chiral self-recognition in aromatic dimers of styrene oxide revealed by rotational spectroscopy

**DOI:** 10.1038/s42004-021-00468-4

**Published:** 2021-03-05

**Authors:** Sérgio R. Domingos, Cristóbal Pérez, Nora M. Kreienborg, Christian Merten, Melanie Schnell

**Affiliations:** 1 Deutsches Elektronen-Synchrotron (DESY), Notkestraße 85, Hamburg, 22607 Germany; 2 Ruhr-Universität Bochum, Fakultät für Chemie und Biochemie, Organische Chemie II, Universitätsstraße 150, Bochum, 44801 Germany; 3 Institut für Physikalische Chemie, Christian-Albrechts-Universität zu Kiel, Max-Eyth-Str. 1, Kiel, 24118 Germany; 4 Present Address: CFisUC, Department of Physics, University of Coimbra, Coimbra, 3004-516 Portugal

**Keywords:** Quantum chemistry, Chemical physics, Origin of life

## Abstract

Chiral molecular recognition is a pivotal phenomenon in biomolecular science, governed by subtle balances of intermolecular forces that are difficult to quantify. Non-covalent interactions involving aromatic moieties are particularly important in this realm, as recurring motifs in biomolecular aggregation. In this work, we use high-resolution broadband rotational spectroscopy to probe the dynamic conformational landscape enclosing the self-pairing topologies of styrene oxide, a chiral aromatic system. We reach a definite assignment of four homochiral and two heterochiral dimers using auxiliary quantum chemistry calculations as well as structure-solving methods based on experimental isotopic information. A complete picture of the dimer conformational space is obtained, and plausible routes for conformational relaxation are derived. Molecular structures are discussed in terms of conformational flexibility, the concerted effort of weak intermolecular interactions, and their role in the expression of the molecular fit.

## Abstract (web summary)

Chiral molecular recognition is critical in many natural processes, but probing the conformational landscape of chiral aromatic systems can be challenging. Here, high-resolution broadband rotational spectroscopy data is combined with quantum chemical computations to reveal the dynamic self-pairing topologies of the chiral molecule styrene oxide.

## Introduction

The concept known as chiral molecular recognition, that is, the ability of a chiral molecule to distinguish between the two enantiomers of another molecule, is critical in many natural processes involving aggregation and assembly of large biomolecules^1,2^. This intrinsic property of handedness enables stereoselectivity (chiral discrimination) and is at the basis of established techniques used for identification and separation of chiral species, namely liquid chromatography^3^, nuclear magnetic resonance^4^ and the recently introduced chiral tagging technique using rotational spectroscopy^5^. Molecular recognition is a direct consequence of non-covalent interactions, generally resulting in contrasting differences in the 3D structures of homo- and hetero-configurational aggregates^6–10^. These diastereomeric complexes differ in their stabilities, leading to chiral discrimination. Understanding the intricate balance between the intermolecular interactions at play during molecular aggregation is thus key to augment our knowledge of recognition processes at the molecular scale.

Particularly relevant in this domain of chemical science are aromatic systems, given their abundance in biological environments as stabilisation units in larger macromolecular assemblies^11–14^. In this framework, much effort has been put into understanding the structure and dynamics of the benzene dimer^15–23^. This prototypical system is a base to study aromatic contacts, showing two fundamentally different arrangements that are almost isoenergetic: the tilted T-shaped and the parallel-displaced (PD) dimer. However, the presence of substituents on the aromatic ring, irrespective of their electron withdrawing or donating nature, seems to lead to favoured parallel configurations^24^. This is in line with an apparent prevalence of stacked structures in biological media and in organic crystals^25,26^.

Direct spectroscopic detection of chiral molecular recognition in the gas phase was first reported by the Zehnacker-Rentien and Giardini-Guidoni groups using laser induced fluorescence^27^, hole-burning^28^, and resonance-enhanced multiphoton ionisation^29^ spectroscopies. During the last decade, Zehnacker-Rentien, Suhm and coworkers, and others have expanded these studies using UV-IR and Fourier transform infra-red spectroscopies^30–32^. With the recent insights brought by these experiments on increasingly larger chiral organic molecules, our understanding of chiral recognition has slowly departed from the more simplistic “three-point” interaction picture to pairing models where weak interactions play a decisive act. Moreover, the balance of dispersion and electrostatic interactions needs to be considered if one is to decode the nature of the contact points that mediate the molecular fit and hence chiral recognition^33–36^.

High-resolution rotational spectroscopy has in recent years entered this scope of studies, providing definite structures of weakly bound complexes of chiral molecules formed in supersonic jet environments. Those include the butan-2-ol^37^, propylene oxide^38^, sevoflurane^39^ and tetrahydro-2-furoic acid^40^ dimers, and the hexafluoroisopropanol^41^ and propylene oxide^42^ trimers. Styrene oxide (SO) or phenyl oxirane is one of the simplest chiral aromatic molecules, and the main metabolite of styrene in humans^43^. It has an oxirane unit connected to the aromatic substructure via a carbon single bond, granting it an apparent rigid structure with a single conformer and a permanent dipole moment of ~1.8 Debye.

Here, we report an in-depth structural study centred around chiral self-recognition of SO. We use high-resolution rotational spectroscopy, which provides us definite assignments of complex molecular geometries, including clusters, in the gas phase^44–55^. Because of the direct correspondence between the unique moments of inertia of the molecules and their 3D structures, geometries of cluster topologies can be identified from their rotational spectrum and directly compared with theoretical structure calculations. We thus paired our experimental observations with a multitude of theoretical modelling approaches, including density functional theory, coupled cluster theory, symmetry-adapted perturbation theory, non-covalent interactions and aromaticity calculations. We obtain unambiguous evidence of dimer formation with both homochiral and heterochiral configurations.

The observed dimers portrait distinct pairing motifs depending on the stereochemistry of the monomeric units and the dynamic relative arrangement of aromatic and oxirane subunits. We quantify the balance of intermolecular forces for each topology and rationalise the observed geometric and energetic trends of the dimers with respect to these forces. We find that the aromatic contact in SO can function either as a single-point interaction or as a pseudo-two-point interaction^56^, consequently affecting the energetic balance in a non-trivial manner for homochiral and heterochiral dimers. We explore here the intricacies of this self-pairing, taking into account conformational flexibility and the roles of dispersion and electrostatics.

## Results and discussion

### Dimer conformational space

**Fig. 1:**
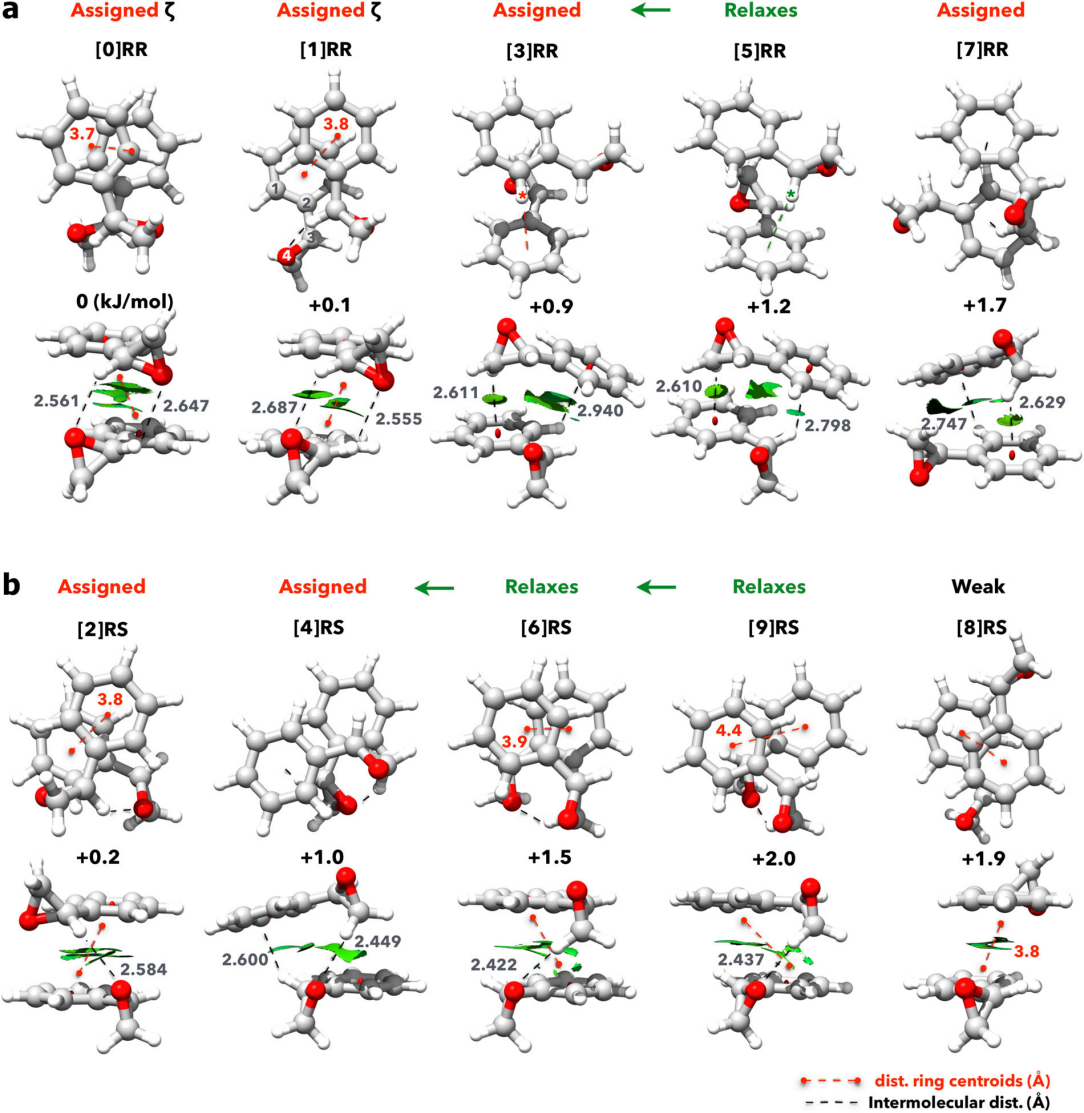
Two views of the molecular geometries of the styrene oxide dimer calculated at the B3LYP-D3(BJ)/def2-TZVP level of theory. For each homochiral (**a**) and heterochiral dimer (**b**), relevant intermolecular interactions (distances given in Å) are depicted and the NCI surfaces plotted. The values shown between the two views are the relative zero-point corrected energies (in kJ mol^−1^) as reported in Table 1. The assigned dimers are highlighted in red, while the ones marked in green are shown to relax to lower energy forms (see section “Relative stability and conformational flexibility”). The *ζ* in dimers [0]RR and [1]RR represents an indirect assignment (see text for further explanation). The asterisk depicted for dimers [3]RR and [5]RR highlights the hydrogen atom that is interacting with the aromatic ring.

In Fig. 1 we show the refined results of the conformational search (see details in the Methods section 4.2.1). All the dimers depicted are geometries that are real local minima, verified based on their harmonic frequencies, with relative energies up to 2 kJ/mol above the predicted global energy minimum. Figure 1a and b depict the homochiral dimers (RR) and heterochiral dimers (RS), respectively, according to their increasing relative energy, from left to right. The predicted rotational constants and dipole moment components for each dimer are given in the upper panel of Table 1. With a first glance at the different dimer topologies, one can readily group them in distinct categories. Primarily, we can sort them based on the relative arrangement of aromatic subunits: dimers [0]RR, [1]RR, [2]RS, [6]RS, [8]RS and [9]RS have their aromatic substructure arranged in a stacked form, similar to that of the PD benzene dimer, while the remaining dimers use CH−*π* contacts, resembling a tilted T-shaped benzene dimer. Additionally, we will take into account the relative orientation of the oxygen lone pairs of each oxirane subunit with respect to the dimer core, that is, dimers [0]RR, [1]RR, [2]RS and [8]RS have both oxygens pointing inwards; dimers [3]RR, [4]RS, [5]RR, [6]RS and [9]RS have one oxygen pointing inwards and another outwards; finally, dimer [7]RR has both oxygens pointing outwards. Solely and qualitatively based on this grouping, theory seems to predict the aromatically stacked dimers with participating oxygens to be the most favourable geometries for dimer formation. We note that topologies comprising stacking of the oxirane unit with the phenyl ring were also predicted, but those are much higher in energy (>10 kJ/mol) and were not considered further in this work. Moreover, the chirality of each monomer, manifested via the orientation of the oxirane moieties, reflects strongly on the number of available binding sites. For example, for the homochiral dimer [0]RR, two CH−O contacts are possible while for the heterochiral dimer [2]RS, a single CH−O contact takes place (Fig. 1). Relevant intermolecular contacts are identified by the non-covalent interactions (NCI)^57,58^ analysis (green surfaces in Fig. 1). This theoretical strategy has been developed to visualise intra- and intermolecular interactions from the topological analysis of the electron density and of its reduced gradient, and it has been successfully used to study chiral molecular recognition^59^. Here, all *π*−*π*, CH−*π*, and CH−O contacts are clearly identified with the colour green, which promptly indicates a predominance of weak interactions in these aggregation motifs. Typically, NCI surfaces will assist in the mapping of strong electrostatic (blue), van der Waals (green) and repulsive (red) forces, and are here used to support our structural analysis and identification of contact points discussed in the following sections.

**Table 1:** Calculated spectroscopic parameters for homochiral and heterochiral dimers of styrene oxide.

	[0]RR	[1]RR	[3]RR	[5]RR	[7]RR	[2]RS	[4]RS	[6]RS	[8]RS	[9]RS
A/MHz	488.65	576.58	517.06	535.83	512.14	477.33	543.67	488.65	634.72	517.33
B/MHz	442.62	392.81	358.59	364.28	370.22	405.81	336.13	399.56	357.27	358.60
C/MHz	340.22	280.20	324.15	304.66	307.60	374.47	320.76	354.73	274.40	334.80
∣*μ*_a_∣/D	0.0	0.6	1.7	1.5	1.2	0.5	2.1	3.5	0.2	3.0
∣*μ*_b_∣/D	0.0	0.5	1.3	2.2	0.9	0.6	2.6	0.6	0.2	1.4
∣*μ*_c_∣/D	0.0	0.1	2.4	1.3	1.6	1.2	0.4	0.5	0.1	1.0
Δ*E*_ZPVE_/kJ mol^−1^	0	+0.1	+0.9	+1.2	+1.7	+0.2	+1.0	+1.5	+1.9	+2.0
Δ*E*_CCSD(T)_/kJ mol^−1^	0	+1.1	+2.9	+3.2	+3.4	+0.8	+3.5	+3.6	+3.3	+4.4
*E*_CP_/kJ mol^−1^	−29.1	−29.4	−29.3	−29.0	−28.7	−28.8	−28.6	−28.0	−27.4	−27.5
Δ*E*_elec_/kJ mol^−1^	−22.3	−23.6	−22.3	−22.0	−21.0	−23.0	−21.8	−22.0	−21.9	−22.1
Δ*E*_exch_/kJ mol^−1^	+49.7	+48.4	+44.7	+44.6	+48.0	+51.5	+44.2	+51.8	+49.5	+47.0
Δ*E*_ind_/kJ mol^−1^	−5.9	−6.5	−6.2	−6.4	−5.8	−6.1	−6.4	−6.4	−5.9	−6.0
Δ*E*_disp_/kJ mol^−1^	−52.7	−49.6	−47.2	−46.2	−49.5	−53.1	−45.5	−51.9	−49.7	−47.3
Δ*E*_total_/kJ mol^−1^	−31.3	−31.3	−31.0	−29.9	−28.3	−30.8	−29.6	−28.4	−27.9	−28.5

Predicted rotational constants, dipole moment components and relative zero-point corrected electronic energies using the B3LYP-D3(BJ)/def2-TZVP level of theory; single-point energies ΔE_CCSD(T)_ calculated using the DLPNO-CCSD(T) method; counterpoise-corrected interaction energies (*E*_CP_); energy (kJ mol^−1^) decomposition obtained from an SAPT2+(3)/aug-cc-pVDZ calculation on all dimers using Psi4 (ref. ^80^). Δ*E*_elec_ is the electrostatic energy, Δ*E*_exch_ represents the repulsion due to exchange, Δ*E*_ind_ is the induction energy accounting for charge transfer interactions and Δ*E*_disp_ is the energy contribution from dispersion interactions.

### Broadband rotational spectroscopy

**Fig. 2:**
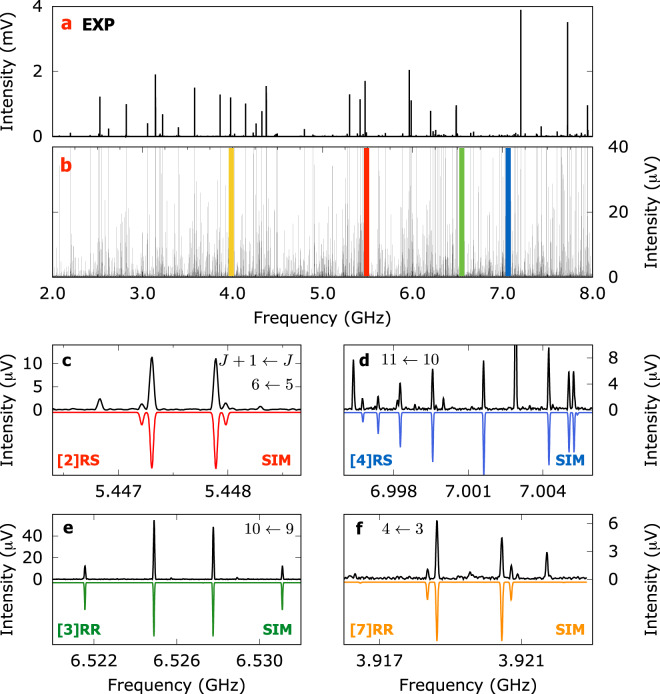
Rotational spectra of SO in the 2–8 GHz range. **a** Fourier transform broadband microwave spectrum obtained after co-adding and averaging 4M FIDs (15 h of measurement time). **b** Full frequency range vertical zoom highlighting relevant spectral portions. **c**–**f** Experimental spectral portions (upper traces, in black) and simulations (lower traces) obtained from the fitted spectroscopic parameters for dimers [2]RS (**c** red), [4]RS (**d** blue), [3]RR (**e** green) and [7]RR (**f** yellow).

Figure 2a displays the high-resolution microwave spectrum of a racemic mixture of SO enantiomers measured in the Hamburg COMPACT spectrometer^60^. The intense lines (1–4 mV) observed in panel a are all assigned to the SO monomer^61^. Figure 2b shows a full range vertical zoom (0–40 μV) to reveal the weaker rotational transitions that are present. The outstanding spectral density is evident as the spectrum contains thousands of well-resolved rotational lines (2203 lines with SNR above 3:1). Four coloured markers are depicted in panel b to locate the frequency regions shown in panels c–f. Panels c and d show two spectral regions, where fitted rotational patterns are tentatively assigned to heterochiral dimers [2]RS and [4]RS, respectively. This assignment is based on a comparison of experimental and calculated rotational constants (see Tables 1 and 2) also considering the relative magnitudes of the dipole moment components. Similarly, in panels e and f, the fitted rotational structures are tentatively assigned to homochiral dimers [3]RR and [7]RR, respectively. The assigned rotational transitions ${J}_{{K}_{{\mathrm{a}}}{K}_{{\mathrm{c}}}}\leftarrow {J}_{K^{\prime} K^{\prime} }^{\prime}$ are denoted as *J*, *K*_a_ and *K*_c_, with *J* being the rotational angular momentum quantum number and *K*_a_ and *K*_c_ being the projections of *J* onto the principal axes at the prolate and oblate symmetric top limits, respectively. The fitted spectroscopic parameters, that is, primary rotational constants (A,B,C) and centrifugal distortion constants, were obtained via a recurrent fit of the semi-rigid rotor Hamiltonian in the A-reduced form using PGOPHER^62^. Lists of the assigned rotational transitions for all the molecular species discussed in this work are given in Supplementary Tables 4–24.

**Table 2:** Observed spectroscopic parameters for homochiral and heterochiral dimers of styrene oxide.

	[0]RR^a^	[1]RR^b^	[2]RS	[3]RR	[4]RS	[7]RR
A/MHz	378.28285(20)	572.181(12)	476.41175(21)	519.837962(47)	565.132480(93)	512.20992(15)
B/MHz	330.824777(86)	386.3259(45)	399.70525(14)	350.271983(38)	327.768274(72)	360.88847(11)
C/MHz	249.305701(65)	276.86249(37)	371.57747(16)	322.618306(39)	307.883752(74)	302.976455(82)
*D*_*K*_/kHz	−0.3622(31)	–	−0.1967(63)	−0.16430(21)	0.1576(11)	−0.0235(37)
*D*_*JK*_/kHz	0.4531(14)	–	0.1363(66)	0.15629(25)	−0.0458(13)	−0.0180(28)
*D*_*J*_/kHz	0.01625(30)	0.03487(93)	0.0758(14)	0.07994(16)	0.07401(34)	0.06366(55)
*δ*_*K*_/kHz	0.23156(74)	–	0.3045(86)	–	0.0455(96)	0.1565(36)
*δ*_*J*_/kHz	−0.00562(16)	–	0.00558(78)	–	0.00293(19)	0.00636(27)
∣*μ*_a_∣	y	y	n	y	y	y
∣*μ*_b_∣	n	y	y	y	y	y
∣*μ*_c_∣	n	n	y	y	y	y
*N*	148	^b^	159	470	342	148
*σ*/kHz	3.7	6.8	5.6	4.7	4.9	3.1

Primary rotational constants (A,B,C) are given in MHz, and quartic centrifugal distortion constants are given in kHz. The errors for the measured values are standard errors. *N* is the number of lines included in the fit, and *σ* is the standard deviation of the fit. The experimental frequency accuracy is 25 kHz.

^a^Rotational constants and centrifugal distortion constants for the global minimum geometry of the homochiral dimer of 2-(4-fluorophenyl)oxirane, which is predicted to be structurally equivalent to [0]RR (Supplementary Fig. 3).

^b^The rotational constants shown for the [1]RR dimer correspond to a partial fit to the centre frequencies of a splitting pattern tentatively assigned to a large-amplitude motion inverting the a- and b-type dipole moment components.

**Fig. 3:**
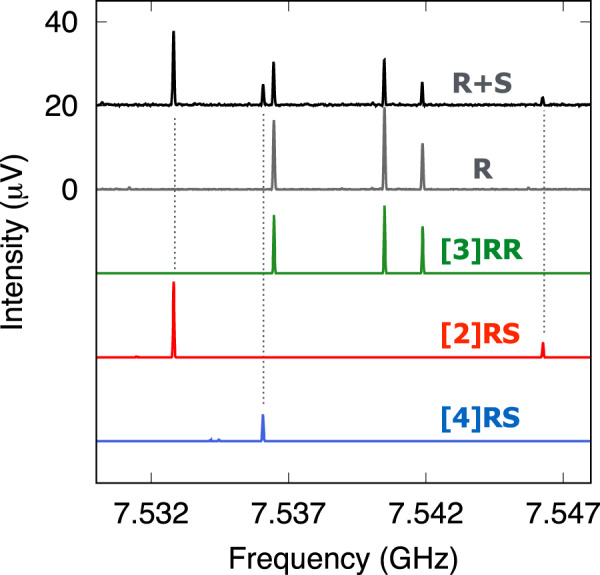
Rotational spectra for racemic and enantiopure SO. Portion of the broadband spectra for racemic (R+S, black trace) and enantioenriched (R, grey trace) samples. Simulations based on the fitted spectroscopic parameters of homochiral [3]RR (green) and heterochiral ([2]RS (red), [4]RS (blue)) dimers are plotted below.

To unambiguously confirm our assignments of homochiral and heterochiral dimers, we used a sample of R enantiomer exclusively and performed an auxiliary broadband spectroscopy measurement. Naturally, the single-enantiomer experiment will constrain the formation of dimers to homochiral aggregates only. A spectral comparison of both racemic (R+S) and pure (R) spectra will consequently allow us to evaluate the chirality of the dimers and definitely assign homochiral and heterochiral species. In Fig. 3 we show a portion of the broadband spectrum for R+S (upper trace, in black) and R (second trace, in grey) samples. The coloured traces below show simulations based on the fitted spectroscopic parameters given in Table 2 for dimers [2]RS, [3]RR and [4]RS. As it now becomes clear, the assigned rotational transitions to dimer [3]RR are present in both R+S and R spectra, confirming its assignment as homoconfigurational. On the other hand, the rotational lines plotted for dimers [2]RS and [4]RS are only visible in the racemic (R+S) spectrum, thus confirming the hetero-configurational nature of the aggregates. The same verification can be made for homochiral dimer [7]RR as is shown in Supplementary Fig. 1.

Whilst a definite assignment of the energetically most favourable heterochiral dimer is reached, the homochiral aggregates have more peculiar properties: the homochiral dimer [1]RR is practically isoenergetic with [0]RR based on the DFT predictions shown in Table 1 and depicted in Fig. 1a. To ascertain this apparent degeneracy we performed DLPNO−CCSD(T) calculations for all homochiral and heterochiral dimers (see details in the Methods section 4.2.1). The results are shown in Table 1 and readily suggest a somewhat larger energy difference (~1 kJ/mol) between dimer [0]RR and [1]RR, favouring the former as the global minimum. The geometrical similarities between the two homochiral dimers are obvious as both assemble in a stacked configuration of phenyl rings. Moreover, both dimers utilise their oxirane subunits to reach two cooperative CH−O interactions with similar binding strengths (see Fig. 1a). Importantly, we find that in the [0]RR dimer, the monomers are arranged in a crossed shape, consequently cancelling out the permanent dipole moment. On the other hand, in the [1]RR dimer, the monomers aggregate in a collinear arrangement, generating a measurable dipole moment for a- and b-type transitions (see Table 1).

Surprisingly, we could not, in a first instance, assign the [1]RR dimer in the experimental spectrum. That is due to a noticeable splitting pattern of the rotational transitions that emerges near our tentative assignments based on the calculated rotational constants for [1]RR (Table 1). A relevant portion of the spectrum is shown in Supplementary Fig. 2. The splitting pattern is present in both racemic (R+S) and enantiopure (R) spectra, confirming we are dealing with a homochiral topology. A preliminary fit using the centre frequencies of the observed patterns is given in Table 2 and allows us to confidently assign the [1]RR dimer by comparison with the DFT predictions. The collinear arrangement of SO monomers entails a large-amplitude motion that inverts the signs of both a- and b-type dipole moments. A final fit of the observed splitting pattern and governing dynamics will be reported elsewhere as it is out of the scope of this study.

Given the lack of a measurable permanent dipole moment, the homochiral dimer [0]RR could not be observed directly in the spectrum (Fig. 2). To surpass this cancellation of dipole moments, we performed another auxiliary broadband measurement using a sample of racemic 2-(4-fluorophenyl)oxirane (F-SO). F-SO is structurally similar to SO, having a fluorine atom in the *para* position (see Supplementary Fig. 3). Calculated rotational constants and dipole moment components for the singly and doubly fluorine-substituted [0]RR dimers of SO are given in Supplementary Table 1. A comparison of molecular structures for the [0]RR and F-[0]RR dimers is shown in Supplementary Fig. 3, displaying a high level of geometrical similarity. Moreover, a conformational search for F-SO dimers, similar to that performed for SO, reveals that the 2F-[0]RR dimer topology is also the global energy minimum, despite the electron withdrawing character of F.

For comparison purposes, complementary SAPT calculations on the 2-(4-fluorophenyl)oxirane dimer show that a small variation in the balance of dispersion and electrostatic contributions occurs with respect to SO, favouring electrostatics with an increase of 2–3% (see Supplementary Table 2). The effect of aromatic ring fluorination on CH-*π* interactions is more extensively discussed in a series of papers addressing the benzene-acetylene^63^, fluorobenzene-acetylene^64^ and difluorobenzene-acetylene^65^ complexes. We note that while double fluorination was shown to significantly alter the geometry of the benzene-acetylene complex, here the parallel-displaced geometry of the [0]RR dimer is equivalent in SO and in 2-(4-fluorophenyl)oxirane. Based on these predictions, we successfully fitted the rotational spectra for the 2F-[0]RR dimer (see Table 2) and the geometrically equivalent singly substituted dimers. Therefore, we can confidently assign − without reasonable doubt − the [0]RR homochiral dimer as the global energy minimum geometry in our study.

### Relative stability and conformational flexibility

We have now reached direct assignment of a total of four dimers (two homochiral and two heterochiral dimers), and two additional homochiral dimers that were indirectly assigned, [0]RR and [1]RR. The remaining four predicted topologies (one homochiral and three heterochiral) could not be observed and will now be addressed considering potential conformational relaxation pathways. Interconversion between conformers separated by low energy barriers − including clusters − is expected to occur within the experimental environment created in a supersonic expansion. A closer look at dimers [3]RR and [5]RR readily reveals their structural similarity, which also transpires from their rotational constants (Table 1), making the assignment non-trivial. The contrasting difference between them is highlighted in Fig. 1a with an asterisk. In the [5]RR dimer, the hydrogen atom at the chiral carbon atom makes the aromatic contact with the phenyl ring, while in the [3]RR dimer, the CH−*π* interaction involves both phenyl rings, as in a pure T-shaped benzene dimer topology.

**Fig. 4:**
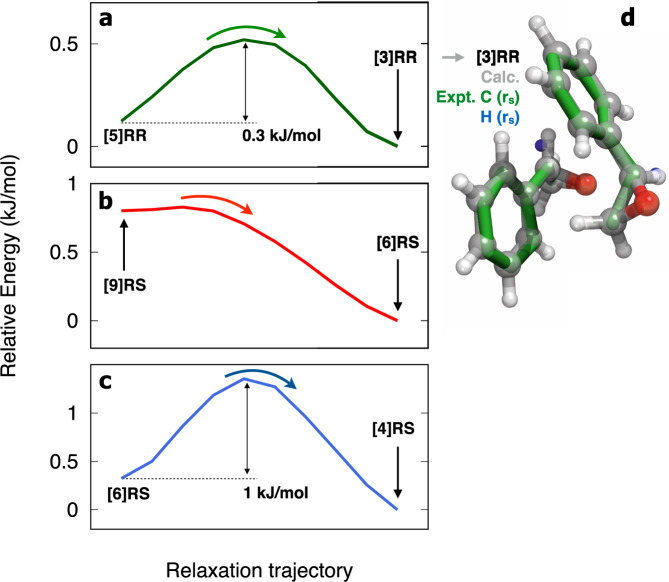
Relaxation trajectories connecting the local minima of the relevant SO dimers. The calculated pathways were computed using the Nudged Elastic Band method at the B3LYP/def2-TZVP level of theory as implemented in ORCA. **a** Trajectory [5]RR → [3]RR, **b** trajectory [9]RS → [6]RS, **c** trajectory [6]RS → [4]RS and **d** overlay of experimental (r_*s*_) and calculated geometries of the [3]RR dimer.

To prove that dimer [5]RR is absent due to conformational relaxation into [3]RR, a series of auxiliary studies were performed. Firstly, we calculated potential conformational relaxation pathways for the [5]RR → [3]RR interconversion. In Fig. 4a we show a relaxation trajectory obtained using the Nudged Elastic Band method^66^, with a predicted energy barrier of ~0.3 kJ/mol (25 cm^−1^), suggesting a favourable relaxation pathway from [5]RR into [3]RR. Taking into account the predicted dipole moment components of each dimer (see Table 1), we note that the relative magnitude of a and b components is inverted for the two dimers. This feature can be used as additional information to verify our assignment. In Supplementary Fig. 4 we show two portions of the spectrum where groups of [baab] transitions are highlighted and compared to simulations where either *μ*_*b*_ > *μ*_*a*_ or *μ*_*a*_ > *μ*_*b*_. The observed pattern clearly matches the predicted one for the [3]RR dimer, with *μ*_*a*_ > *μ*_*b*_. Finally, and to obtain definite evidence of the dimer geometry [3]RR detected in the spectrum, we assigned the rotational transitions arising from the corresponding singly substituted ^13^C isotopologs in natural abundance. All the 16 carbon atom positions were then derived using the Kraitchman equations (*r*_*s*_-method)^67,68^ (see Fig. 4d). The experimental match to the calculated [3]RR geometry is clear. Furthermore, and considering the close similarity between geometries [3]RR and [5]RR, we performed an additional broadband measurement using a sample of deuterated SO (d-SO) mixed with normal SO in a ratio of ~1:1 to extract the spectra of the singly substituted isotopolog (H → D) corresponding to the hydrogen atom participating in the aromatic embedding (see the Supplementary Methods). The fitted rotational constants for both isotopic species are given in Supplementary Table 3. The location of the relevant hydrogen atom is revealed with respect to the centre of mass of the dimer, further confirming the assignment of the [3]RR topology (see Fig. 4d and Supplementary Fig. 5).

Due to their structural similarity, an analogous conclusion can be reached for the absent heterochiral dimers [9]RS and [6]RS. In fact, the calculated conformational relaxation trajectory reveals that [9]RS can be converted into [6]RS via an energetically costless displacement of the phenyl ring (see Fig. 4b). This would explain the absence of [9]RS from the spectrum. Following this path, in Fig. 4c we show the calculated trajectory to relax dimer [6]RS into [4]RS. With a predicted interconversion barrier of 1 kJ/mol, this pathway suggests further relaxation into dimer [4]RS. We note that [6]RS is structurally similar to [2]RS as it presents the same type of intermolecular contacts (stacked configuration with one additional CH−O interaction). Yet, here the weak hydrogen-bond donor is the CH_2_ group, resulting in a single set of oxygen lone pairs pointing inwards the dimer core and consequently a less stable topology. Finally, while structurally distinctive from all others, [8]RS has an opposite arrangement of ether subunits, resulting in cancellation of dipole moments and rendering it undetectable with pure rotational spectroscopy.

**Fig. 5:**
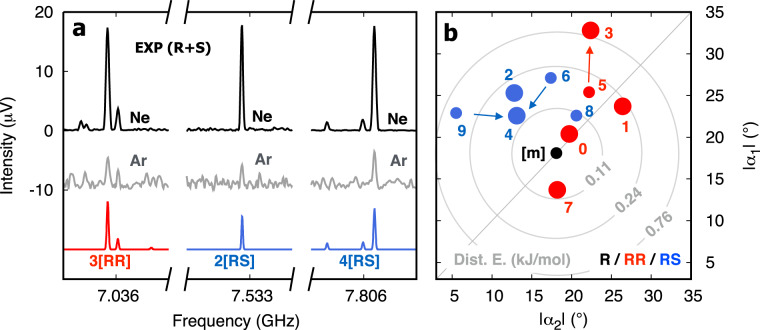
Relative stabilities and distortion energies of the relevant SO dimers. **a** Portions of the broadband spectra using neon (black trace) and argon (grey trace) as seeding gas. The coloured traces are simulations based of the fitted spectroscopic parameters given in Table 2. **b** Scatter plot depicting calculated dihedral angles (*α* = C1 − C2 − C3 − O4, as in [1]RR in Fig. 1) for the SO monomer (in black), homochiral (in red) and heterochiral (in blue) dimers. The larger dots represent observed dimers. The smaller dots indicate non-observed dimers, with arrows pointing to proposed relaxation trajectories. The concentric traces indicate the energy increase due to torsion of the dihedral angle at the monomer geometry.

To expand further our understanding of the conformational landscape of the SO dimer, we will now discuss the relative stability of the observed species. For that we performed an experiment using a mixture of SO enantiomers using similar experimental conditions as shown in Fig. 2, except we used argon as a carrier gas instead of neon. It is known that the larger collision energy provided by heavier gases during supersonic jet expansions facilitate isomerization of higher energy species into lower energy ones^20,69^. An argon-seeded supersonic expansion is thus expected to favour the lower-energy conformers of the SO dimer. As introduced earlier in this work, we can group the SO dimers in different categories depending on their relative arrangement of aromatic units and of the oxygen atoms in the oxirane subunits. Dimers [3]RR, [2]RS and [4]RS are all different in this sense, showcasing distinct intermolecular contact schemes as verified by the NCI analysis: dual CH−*π* docking; *π*−*π* stacking with single CH−O docking; and one CH−*π* paired with one CH−O interaction, respectively. In Fig. 5a, we show three portions of the broadband spectra (R+S) using Ne (upper trace, in black) and Ar (grey trace) as carrier gases. The coloured traces are the simulated spectra for dimers [3]RR, [2]RS and [4]RS using the fitted spectroscopic parameters reported in Table 2. The plot nicely confirms the relative stability of each subgroup of dimers, as rotational lines for all three species are observed in both neon- and argon-seeded spectra. This strongly suggests that the homochiral dimer [3]RR does not relax into any of the two more stable stacked homochiral dimers. Similarly, we learn that the heterochiral dimer [4]RS does not convert into the more stable dimer [2]RS, even with the increased collision energy. As for dimer [1]RR, whilst structurally distinct from the others (*π*−*π* stacking with double CH−O docking), an assignment was not possible in the argon-seeded spectrum. This could be for one of two reasons: either a statistical depletion due to a significantly reduced SNR in the argon spectrum; or conformational relaxation occurs and dimer [1]RR converts into the more stable non-polar [0]RR dimer, which is undetectable. Similar conclusions can be drawn for the absence of [7]RR in the Ar spectrum.

Another interesting feature, closely connected to the internal stability of the observed dimers, is presented in Fig. 5b, where we have produced a scatter plot depicting the dihedral coordinate *α* along the C−C bond for all dimers and the monomer of SO. In addition, concentric traces show the energy variations due to distortion of the phenyl ring from the equilibrium position. These were calculated from a relaxed scan of the dihedral angle along the single bond for the monomer of SO. Remarkably, the dihedral coordinates of the SO monomers are noticeably loosened to promote dimerisation in both homochiral and heterochiral species. The extent of the reshaping varies according to the type of dimer. For example, the formation of homochiral dimer [0]RR is favoured with minimal changes in the dihedral coordinates ([m] → 0 in Fig. 5b). However, the most stable heterochiral dimer, [2]RS, is only reached by overcoming clear dihedral changes ([m] → 2 in Fig. 5b). Dimer [3]RR shows the most pronounced dihedral adjustment of ~15^∘^, which translates to an energy increase of ~0.7 kJ/mol with respect to the equilibrium structure of the monomer. Considering the extent of the reshaping for the heterochiral dimer [2]RS, the small energy differences predicted between [0]RR and [2]RS, 0.2 kJ/mol and 0.8 kJ/mol for DFT and CCSD(T) methods, respectively, appeal to further discussion.

### Tethering effects on the energy and aromaticity

The energetic decomposition captured in the SAPT calculations (lower panel of Table 1) indicates similar contributions of electrostatic (28%) and dispersion (65%) to the binding energy in both [0]RR and [2]RS dimers. Considering that [0]RR has two oxirane contact points, while [2]RS has only one, this analogous balance would suggest that the highly symmetric dual CH−O interaction ([0]RR) introduces a favourable pairing for the oxirane subunits, forcing the rings out of their optimal arrangement (a PD-like geometry). Holding a single CH−O contact in place, [2]RS has, in principle, more flexibility for the aromatic units to stack in a more efficient arrangement. To exclude other contributions and verify our hypothesis, we performed an in silico mutation (phenyl → H) and recalculated the SAPT energetic contributions for both dimers. The results, shown in Table 3, are clear: the total binding energy coming solely from the oxirane units in the [0]RR dimer is superior by a factor 2 compared with that of dimer [2]RS, showing very similar electrostatic and dispersion contributions (within 1%) in both cases. The oxirane pair in the [2]RS dimer is held more prominently by dispersion energy, at the cost of a weakened electrostatic interaction (*E*_disp_ − *E*_elec_ ~ 10%).

**Table 3:** Predicted SAPT energy decomposition for the interaction of oxirane subunits.

	[0]RR	[0]RR-oxirane	[2]RS	[2]RS-oxirane
Elec. (%)	28	43	28	40
Ind. (%)	7	11	7	12
Disp. (%)	65	46	65	48
Total (kJ/mol)	−81	−27	−82	−13

Contributions to the overall interaction energy derived from the SAPT calculations for the in silico (C_6_H_6_→H) mutation in the [0]RR and [2]RS dimer topologies.

These results are consistent with the calculated aromaticity parameters. Using the calculated nucleus-independent chemical shifts (NICS)^70^ for the [0]RR, [2]RS and PD dimers, we produced predictions for the absolute magnetic shielding at the core of each dimer (mid point connecting ring centroids). Using the values of the PD benzene dimer as a reference for optimal stacking (−13.16), we find that the [2]RS dimer (−13.14) presents a more favourable aromatic stacking than the [0]RR dimer (−11.86). All together, these observations indicate that the quasi-optimal self pairing of [2]RS is only surpassed in stability when the hydrogen-bond dyad is formed for the homochiral dimer [0]RR, distorting an optimal stacking configuration, yet adding binding energy via the two electrostatic contacts. This aggregation motif for SO is only possible in a complete homochiral environment.

## Conclusions

The complex conformational space of the styrene oxide dimer is uncovered here using high-resolution rotational spectroscopy in combination with advanced quantum chemistry calculations. Homochiral and heterochiral dimers were observed and their topologies discussed in terms of the prevailing intermolecular interactions and their role in mediating dimerisation. We found that overall, SO dimer formation is strongly driven by dispersion interactions, either with stacked or T-shaped templates. These two distinct pairing topologies, often employed to address the benzene dimer, are used here to separate the SO dimers into conformational families. The internal stability of these subgroups is investigated using calculated relaxation pathways as well as complementary experiments using a heavier carrier gas in the supersonic jet.

Our study reveals that the most stable homochiral aggregate [0]RR has a subtle energetic advantage because of a combination of both *π*−*π* stacking and optimal pairing of oxirane subunits due to their equal handedness. The most stable heterochiral dimer [2]RS forms in a highly efficient stacking arrangement of the two aromatic rings, on equal footing with that of the stacked benzene dimer. This optimal arrangement of aromatic rings is a consequence of the single CH−O interaction at play, facilitating a favourable aromatic contact. This scenario is possible because of the opposite chirality of the monomers, and it comes with only a small energetic cost. The source of this energetic balance is further explored taking into account indicators of aromaticity and a proper decomposition of energetic contributions laid out by the symmetry-adapted perturbation theory calculations. These theoretical indicators seem to suggest that from an energetic perspective, the gain via the additional CH−O interaction in the homochiral dimer [0]RR evens out with the loss of optimal aromatic arrangement of rings. This idea is consistent with the complementary set of counterpoise-corrected calculations on the stacked dimers, which show only small differences in binding energy between homochiral and heterochiral dimers. Moreover, the relative stability studies performed here unveil a high degree of conformational readjustment of the native monomers, indicating a tendency to induce the fit to maximise the stacking. This dynamic character of the self-pairing emerges from the ability of the aromatic plane to rotate with respect to the oxirane unit at a low energetic cost. Several dimer species are thus formed and observed in the rotational spectrum despite the conformational relaxation paths identified.

Putting these observations into perspective with the previous work of Su et al.^38^ on the propylene oxide (PO) dimer, we find a few interesting aspects that deserve attention. PO has a methyl group instead of the phenyl ring present in SO. In the aggregation picture of PO, the authors report that the 10 most stable dimers are held together by four weak hydrogen bonds. Of note is that in all of these predicted species, six of which are detected experimentally, the methyl group is directly involved in the expression of the molecular recognition. This feature is in line with our observations for SO, where the aromatic ring is always at play for the dimerisation. Particularly relevant here is that not only the aromatic group takes the leading role regulating the aggregation, but it is the tethering of the rings that dictates the formation of the most stable species predicted and observed experimentally. This type of self-pairing is naturally not possible for PO.

In summary, the dynamic conformational landscape for the dimerisation of styrene oxide is presented. The lack of dominant hydrogen bonding donor and acceptor groups makes SO a particularly suited system to carefully explore the balance of weaker intermolecular interactions, and it may serve as a blueprint to explore other classes of chiral aromatic systems. In a nutshell, a dispersion-driven primary contact (tethering) is made between the two aromatic subunits, establishing either a stacked or a T-shaped topology. This step is presumed to take a lead, followed by formation of the secondary interactions (mainly represented here by the oxirane CH−O contacts) until an expression of the molecular fit is reached and chiral recognition is established.

## Methods

### Experimental

#### Broadband rotational spectroscopy

Samples of (R)-(+)-SO and (S)-(−)-SO were purchased from Sigma Aldrich (97% and 98% optical purity, respectively) and used without further purification. All experiments reported hereon were performed using the Hamburg COMPACT spectrometer^60^, which is a chirped-pulse Fourier transform microwave spectrometer^71^. A cold molecular jet introduces the SO molecules into the vacuum chamber at rotational temperatures below 2 K. For that, a pulsed nozzle (Parker General Valve Series 9) operating at 9 Hz with a constant flow of Ne or Ar at stagnation pressures of 3 bars is used. The neat liquid samples are introduced in a reservoir directly at the nozzle and heated to 80 ^∘^C to create sufficient vapour pressure. A 4-μs chirp spanning 2–8 GHz is generated in an arbitrary wave-form generator (AWG) and amplified in a 300-W travelling wave tube (TWT) amplifier. The chirped pulse is then broadcasted into the chamber using a microwave horn antenna. The molecular ensemble absorbs the energy from the microwave electric field and is induced to rotate coherently in phase with the incident radiation. Microwave emission is then captured in a second horn antenna as free induction decay (FID). Furthermore, to improve data collection speed and minimise sample consumption, the “fast-frame” option of the digital oscilloscope is used: eight back-to-back chirps excite each gas pulse, and their corresponding FIDs are recorded, co-added, and averaged. In practical terms this measurement scheme results in an effective repetition rate of 72 Hz.

### Theoretical

#### Conformational search and quantum chemistry calculations

To locate the local energy minima for homochiral and heterochiral dimer configurations of SO, two different conformational search methods were used and their results cross-checked against each other. Firstly, the ABCluster algorithm^72^ was used, where the conformations of subunits are constrained thus preventing any structural reshaping to occur to facilitate aggregation. A total of 60 dimers were produced in two separate runs (30 homochiral dimers and 30 heterochiral dimers). All 60 dimer geometries were further optimised using high-level density functional theory (DFT) calculations using ORCA^73,74^. For all predicted structures, the B3LYP-D3(BJ) functional and the def2-TZVP basis set was used. Secondly, we performed a simulated annealing conformational search using the extended tight-binding semi-empirical programme package, XTB^75–77^. Here, the monomeric subunits are allowed to relax and adapt their conformation to facilitate the interlock while forming dimers. A total of 80 dimers were produced, including homochiral and heterochiral forms. These were all further optimised using the same high-level DFT methods employed for the ABCluster dimers.

Single-point energy calculations were performed using the domain-based local pair-natural orbital coupled cluster perturbative triple-excitations (DLPNO-CCSD(T)) method and the def2-TZVPP basis set with the resolution-of-identity (RIJCOSX) approximation as implemented in ORCA^73,74^. Counterpoise-corrected energies (*E*_CP_) accounting for basis set superposition errors (BSSE) were obtained using the Boys and Bernardi formula^78^. Symmetry-adapted perturbation theory (SAPT)^79^ calculations were carried out at the SAPT2+(3)/aug-cc-pVDZ level of theory using Psi4^80^ to predict the decomposition of the energetic contributions arising from the intermolecular binding forces in the different observed dimers. As an auxiliary tool for visualisation and analysis of the relevant intermolecular contacts at work in the dimer formation, we performed a non-covalent interaction (NCI) analysis using NCIPLOT^57^. The density-gradient maps are plotted using UCSF Chimera using isovalues of 0.12 and density ranges from −6 to +6. The nucleus-independent chemical shifts (NICS), which relate directly to the chemical shift observed in NMR spectroscopy, were calculated using Gaussian 09^81^ as a means to evaluate the aromaticity index for the dimers where the phenyl rings are arranged in a stacked geometry. The reported values for [0]RR and [2]RS deliver the predicted absolute magnetic shielding at the dimer cores, that is, at an equivalent distance between phenyl ring centroids.

## Supplementary information

Supplementary Information

## Data Availability

The data supporting the findings of this study are available within the article and its Supplementary Information file. Other data are available from the authors upon request.
