# Supplementary material for: Dynamic chiral self-recognition in aromatic dimers of styrene oxide revealed by rotational spectroscopy
Source: Commun Chem. 2021 Mar 5;4:32. doi: 10.1038/s42004-021-00468-4 (PMC9814401; doi:10.1038/s42004-021-00468-4)
Supplement: Supplementary file 1 — Supplementary Information [file 42004_2021_468_MOESM1_ESM.pdf]

Supplementary Material

Dynamic chiral self-recognition in aromatic dimers of styrene oxide revealed by rotational spectroscopy

Sérgio R. Domingos[1,‡], Cristóbal Pérez[1], Nora M. Kreienborg[2], Christian Merten[2] and Melanie Schnell[1,3]

1 Deutsches Elektronen-Synchrotron DESY, Notkestraße 85, 22607 Hamburg, Germany

2 Ruhr-Universität Bochum, Fakultät für Chemie und Biochemie, Organische Chemie II, Universitätsstraße 150, 44801 Bochum, Germany

3 Institut für Physikalische Chemie, Christian-Albrechts-Universität zu Kiel, Max-Eyth-Str. 1, 24118 Kiel, Germany

‡ Present address: CFisUC, Department of Physics, University of Coimbra, 3004-516, Coimbra, Portugal

Contents

1 Rotational and structural parameters **3**

1.1 [7]RR . . . 3

1.2 [1]RR . . . 3

1.3 SO / F-SO . . . 4

2 Conformational relaxation **5**

2.1 [5]RR → [3]RR . . . 5

3 Supplementary Methods **5**

3.1 d−SO analysis . . . 5

3.1.1 Synthesis of deuterated SO (d−SO) . . . 5

3.1.2 Rotational parameters: d−isotopologs of SO . . . 6

4 Appendix A: Line lists **7**

4.1 FF-[0]RR . . . 7

4.2 [2]RS 11

4.3 [3]RR 16

4.4 [7]RR 29

4.5 [4]RS 33

4.6 [3]RR singly substituted ^{13}C isotopologs 43

1 Rotational and structural parameters

1.1 [7]RR

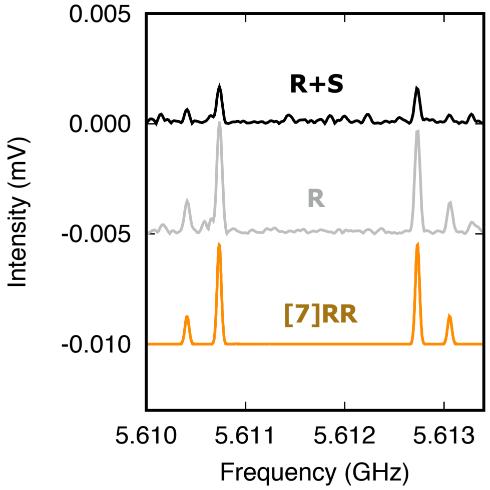

Supplementary Figure 1: Portion of the broadband spectra for racemic (R+S, black trace) and enantioenriched (R, grey trace) samples. A simulation based on the fitted spectroscopic parameters of homochiral [7]RR (yellow) is plotted below

1.2 [1]RR

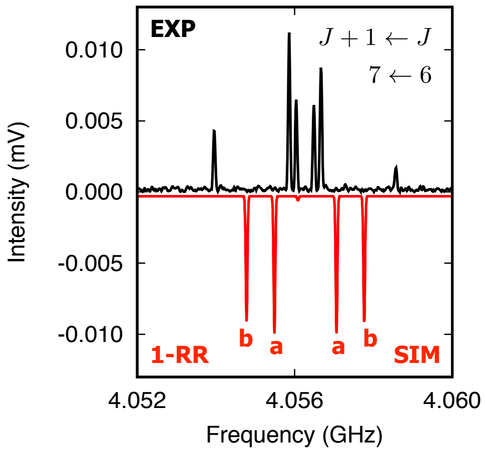

Supplementary Figure 2: Portion of the microwave spectrum of SO showing a partial fit to the center frequencies of a splitting pattern tentatively assigned to a large-amplitude motion leading to inversion of the a- and b-type dipole moment components of dimer [1]RR.

1.3 SO / F-SO

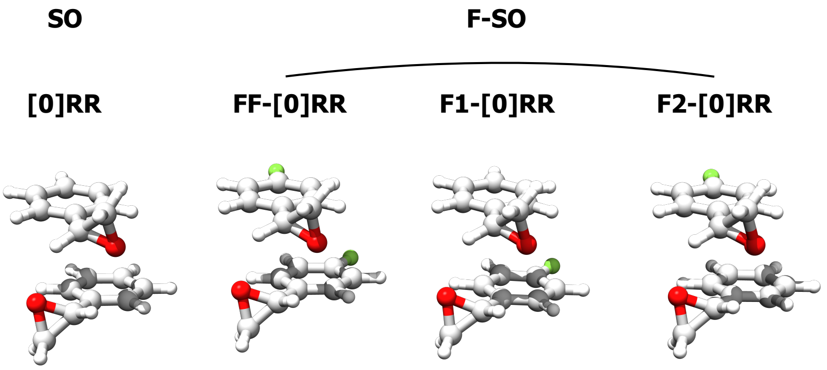

Supplementary Figure 3: Comparison of molecular structures for the SO and F-SO dimers in the homochiral configuration [0]RR. The singly-substitution mixed dimers F1-[0]RR and F2-[0]RR are equivalent.

Supplementary Table 1: Observed and calculated spectroscopic parameters for homochiral dimers of pure 2-(4-fluorophenyl)oxirane (F-SO) and mixed SO and F-SO. Primary (A,B,C) rotational constants are given in MHz and quartic centrifugal distortion constants are given in kHz. The errors for the measured values are standard errors. N is the number of lines included in the fit, and the σ is the standard deviation of the fit. The experimental frequency accuracy is 25 kHz.

	[0]RR-FF		[0]RR-F1/[0]RR-F2	
	Obs.	Theory	Obs.	Theory
A/MHz	378.28285(20)	394.38	425.00416(27)	434.42
B/MHz	330.824777(86)	323.57	383.64192(14)	387.78
C/MHz	249.305701(65)	249.86	284.16058(11)	285.75
D_K/kHz	-0.3622(31)	-	-0.1129(57)	-
D_{JK}/kHz	0.4531(14)	-	0.1522(23)	-
D_J/kHz	0.01625(30)	-	0.11108(59)	-
δ_K/kHz	0.23156(74)	-	0.2724(17)	-
δ_J/kHz	-0.00562(16)	-	0.03531(30)	-
$\|\mu_a\|$	y	2.8	y	1.4
$\|\mu_b\|$	n	0.7	n	0.2
$\|\mu_c\|$	n	0.1	n	0.2
N	148	-	122	-
σ/kHz	3.7	-	4.5	-

Supplementary Table 2: Percentual contributions of dispersion and electrostatic energy to the total SAPT energy at the SAPT2+(3)/aug-cc-pVDZ level of theory for SO and 2-(4-fluorophenyl)oxirane (F-SO) dimers.

	$(SO)_2$	$(F\text{-}SO)_2$	SO-(F-SO)
Dispersion (%)	65.1	62.9	62.5
Electrostatic (%)	27.5	30.2	30.4

2 Conformational relaxation

2.1 [5]RR → [3]RR

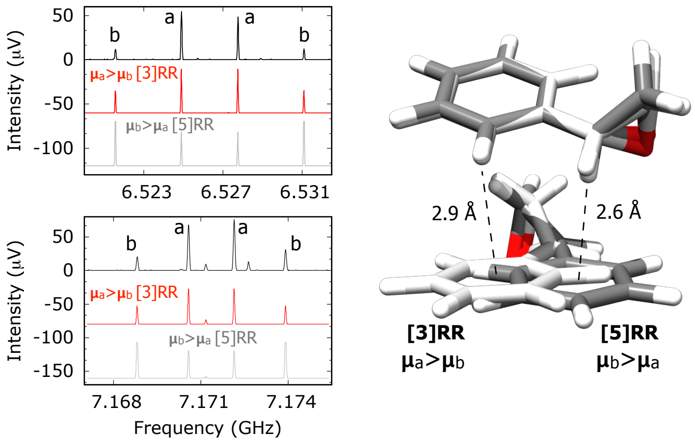

Supplementary Figure 4: Spectral windows showing the microwave spectrum and two [baab] groups of rotational transitions scaled proportionally to the predicted dipole moment components of dimer [3]RR (in red) and [5]RR (in grey). An overlay of relevant geometries is shown on the right as a guide to the eye.

3 Supplementary Methods

3.1 d−SO analysis

3.1.1 Synthesis of deuterated SO (d−SO)

This synthesis has been adapted from the literature.[1] TMEDA (3 ml, 2.3 mg, 20 mmol) was added to a solution of R−SO (0.8 ml, 0.84 mg, 7 mmol) in THF (40 ml) at −105°C under inert gas conditions. Afterwards the solution was treated with sec-BuLi (9 ml, 1.4 M, 12.6 mmol) until the solution turned from colorless to red. After the mixture was stirred for 20 min, methanol-d4 (2 ml, 50 mmol) was added, the mixture was allowed to warm up to room temperature and was stirred overnight. The mixture was diluted with DCM (30 ml) and washed with brine (4x25 ml). The organic layer was dried over MgSO4, the solvent was evaporated under reduced pressure and 0.4 g (3.3 mmol, 47% yield) a pale yellowish oil was obtained. A ratio of 87:13 R−d−SO : R−SO could be determined by 1H NMR spectroscopy. 1H spectroscopy (200 MHz, CDCl3) δ: 2.74 (d, 1 H), 3.08 (d, 1 H), (3.80 (t, 0.15 H)), 7.24 (m, 5H) ppm.

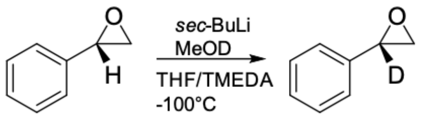

3.1.2 Rotational parameters: d−isotopologs of SO

Supplementary Table 3: Observed and predicted spectroscopic parameters for the D-isotopologs of the SO dimer [3]RR. Primary (A,B,C) rotational constants are given in MHz. Quartic centrifugal distortion constants are locked to the parent species as report in Table I of the main article. The errors for the measured values are standard errors. N is the number of lines included in the fit, and the σ is the standard deviation of the fit. The experimental frequency accuracy is 25 kHz. The scaled constants were predicted using the PROSPE suite of programs.

	[3]RR/dSO(H21)		[3]RR/dSO(H4)	
	Obs.	scaled*	Obs.	scaled*
A/MHz	516.14568(14)	513.87	517.08516(12)	514.75
B/MHz	348.273424(81)	345.72	350.324301(69)	350.23
C/MHz	322.393824(83)	321.01	321.773722(70)	320.67
N	80	-	99	-
σ/kHz	5.8	-	5.8	-

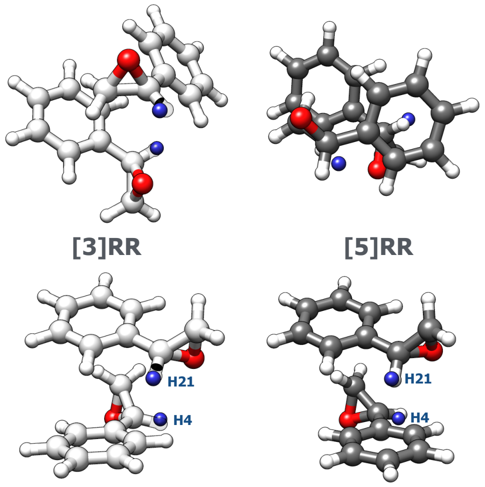

Supplementary Figure 5: Overlay of calculated and r_s structures for dimers [3]RR and [5]RR.

4 Appendix A: Line lists

4.1 FF-[0]RR

Supplementary Table 4: Observed and calculated rotational transitions (MHz) for the FF-[0]RR dimer.

Observed	Calculated	Obs-Calc	J′	K_a′	K_c′	J″	K_a″	K_c″
2095.8301	2095.8339	-0.0038	4	1	4	3	1	3
2098.5950	2098.5961	-0.0011	4	0	4	3	0	3
2278.2556	2278.2559	-0.0003	4	2	3	3	2	2
2325.2714	2325.2708	0.0006	4	1	3	3	1	2
2491.0663	2491.0652	0.0011	4	2	2	3	2	1
2595.4490	2595.4527	-0.0037	5	1	5	4	1	4
2595.9545	2595.9563	-0.0018	5	0	5	4	0	4
2794.2695	2794.2686	0.0009	5	2	4	4	2	3
2811.0345	2811.0373	-0.0028	5	1	4	4	1	3
2947.2836	2947.2793	0.0043	5	3	3	4	3	2
3006.9297	3006.9293	0.0004	5	4	2	4	4	1
3043.6330	3043.6342	-0.0012	5	2	3	4	2	2
3094.2185	3094.2169	0.0016	6	1	6	5	1	5
3094.2998	3094.2988	0.0010	6	0	6	5	0	5
3136.1129	3136.1110	0.0019	5	3	2	4	3	1
3298.3193	3298.3182	0.0011	6	2	5	5	2	4
3483.9046	3483.9055	-0.0009	6	3	4	5	3	3
3536.3044	3536.3043	0.0001	6	2	4	5	2	3
3592.8338	3592.8387	-0.0049	7	0	7	6	0	6
3592.8338	3592.8263	0.0075	7	1	7	6	1	6
3597.6682	3597.6673	0.0009	6	4	3	5	4	2
3619.4365	3619.4345	0.0020	6	5	2	5	5	1
3645.3547	3645.3562	-0.0015	6	5	1	5	5	0
3738.8663	3738.8668	-0.0005	6	3	3	5	3	2
3745.1177	3745.1158	0.0019	6	4	2	5	4	1
3798.0941	3798.0922	0.0019	7	2	6	6	2	5
3798.9346	3798.9330	0.0016	7	1	6	6	1	5
3998.5840	3998.5866	-0.0026	7	3	5	6	3	4
4016.4841	4016.4785	0.0056	7	2	5	6	2	4

4091.4152	4091.4141	0.0011	8	0	8	7	0	7
4091.4152	4091.4123	0.0029	8	1	8	7	1	7
4159.6731	4159.6757	-0.0026	7	4	4	6	4	3
4224.5512	4224.5500	0.0012	7	6	2	6	6	1
4230.1583	4230.1606	-0.0023	7	5	3	6	5	2
4235.0977	4235.0979	-0.0002	7	6	1	6	6	0
4267.0270	4267.0212	0.0058	7	3	4	6	3	3
4296.8004	4296.8020	-0.0016	8	2	7	7	2	6
4296.9535	4296.9547	-0.0012	8	1	7	7	1	6
4398.7911	4398.7938	-0.0027	7	4	3	6	4	2
4501.9501	4501.9478	0.0023	8	3	6	7	3	5
4506.5210	4506.5186	0.0024	8	2	6	7	2	5
4589.9944	4589.9937	0.0007	9	1	9	8	1	8
4589.9944	4589.9939	0.0005	9	0	9	8	0	8
4672.0651	4672.0668	-0.0017	6	5	1	5	3	2
4744.3626	4744.3659	-0.0033	8	3	5	7	3	4
4776.3178	4776.3175	0.0003	6	3	3	5	1	4
4782.7933	4782.7958	-0.0025	14	5	10	13	7	7
4818.0117	4818.0077	0.0040	8	5	4	7	5	3
4825.9800	4825.9814	-0.0014	8	7	2	7	7	1
4829.8975	4829.8971	0.0004	8	7	1	7	7	0
4848.0100	4848.0120	-0.0020	8	6	3	7	6	2
4899.2012	4899.2020	-0.0008	8	6	2	7	6	1
4951.7540	4951.7555	-0.0015	6	5	2	5	3	3
4953.4086	4953.4122	-0.0036	6	4	3	5	2	4
4980.9345	4980.9290	0.0055	8	4	4	7	4	3
5001.3566	5001.3476	0.0090	9	3	7	8	3	6
5002.3359	5002.3380	-0.0021	9	2	7	8	2	6
5018.3009	5018.2996	0.0013	8	5	3	7	5	2
5088.5724	5088.5715	0.0009	10	0	10	9	0	9
5088.5724	5088.5715	0.0009	10	1	10	9	1	9
5204.3580	5204.3581	-0.0001	9	4	6	8	4	5
5221.5061	5221.5079	-0.0018	9	3	6	8	3	5
5293.8467	5293.8447	0.0020	10	2	9	9	2	8
5293.8467	5293.8488	-0.0021	10	1	9	9	1	8
5374.4680	5374.4632	0.0048	9	5	5	8	5	4

5425.8705	5425.8680	0.0025	9	8	2	8	8	1
5427.2256	5427.2337	-0.0081	9	8	1	8	8	0
5455.8283	5455.8286	-0.0003	9	7	3	8	7	2
5457.9723	5457.9748	-0.0025	9	6	4	8	6	3
5479.2341	5479.2399	-0.0058	9	7	2	8	7	1
5483.7741	5483.7789	-0.0048	9	4	5	8	4	4
5499.7969	5499.7991	-0.0022	10	3	8	9	3	7
5499.9903	5499.9921	-0.0018	10	2	8	9	2	7
5587.1462	5587.1444	0.0018	11	1	11	10	1	10
5587.1462	5587.1444	0.0018	11	0	11	10	0	10
5601.3110	5601.3115	-0.0005	9	6	3	8	6	2
5659.1984	5659.1992	-0.0008	9	5	4	8	5	3
5706.4073	5706.4114	-0.0041	10	4	7	9	4	6
5710.8774	5710.8766	0.0008	10	3	7	9	3	6
5792.3722	5792.3726	-0.0004	11	2	10	10	2	9
5792.3722	5792.3732	-0.0010	11	1	10	10	1	9
5950.3535	5950.3549	-0.0014	10	4	6	9	4	5
5998.1520	5998.1349	0.0171	11	3	9	10	3	8
5998.1520	5998.1699	-0.0179	11	2	9	10	2	8
6067.5678	6067.5569	0.0109	10	8	2	9	8	1
6081.5003	6081.4948	0.0055	10	7	4	9	7	3
6085.7153	6085.7112	0.0041	12	0	12	11	0	11
6085.7153	6085.7112	0.0041	12	1	12	11	1	11
6166.8687	6166.8684	0.0003	10	7	3	9	7	2
6205.1654	6205.1604	0.0050	11	4	8	10	4	7
6206.1669	6206.1684	-0.0015	11	3	8	10	3	7
6215.0721	6215.0625	0.0096	10	5	5	9	5	4
6290.9075	6290.9078	-0.0003	12	1	11	11	1	10
6290.9075	6290.9077	-0.0002	12	2	11	11	2	10
6293.4314	6293.4343	-0.0029	10	6	4	9	6	3
6410.9196	6410.9233	-0.0037	11	5	7	10	5	6
6426.4038	6426.4063	-0.0025	11	4	7	10	4	6
6496.5132	6496.5105	0.0027	12	3	10	11	3	9
6496.5132	6496.5165	-0.0033	12	2	10	11	2	9
6584.2632	6584.2710	-0.0078	13	1	13	12	1	12
6584.2632	6584.2710	-0.0078	13	0	13	12	0	12

6590.0783	6590.0813	-0.0030	11	6	6	10	6	5
6655.8556	6655.8522	0.0034	8	7	2	7	5	3
6661.0408	6661.0429	-0.0021	11	9	2	10	9	1
6688.1023	6688.1046	-0.0023	11	7	5	10	7	4
6692.5619	6692.5600	0.0019	11	8	4	10	8	3
6694.8126	6694.8125	0.0001	11	5	6	10	5	5
6703.2186	6703.2168	0.0018	12	4	9	11	4	8
6703.4249	6703.4233	0.0016	12	3	9	11	3	8
6709.0106	6709.0134	-0.0028	8	4	5	7	2	6
6709.2444	6709.2420	0.0024	9	6	3	8	4	4
6735.7025	6735.7047	-0.0022	11	8	3	10	8	2
6789.4417	6789.4450	-0.0033	13	2	12	12	2	11
6789.4417	6789.4450	-0.0033	13	1	12	12	1	11
6884.0415	6884.0393	0.0022	11	7	4	10	7	3
6911.4409	6911.4485	-0.0076	12	5	8	11	5	7
6915.5225	6915.5249	-0.0024	12	4	8	11	4	7
7082.8194	7082.8228	-0.0034	14	1	14	13	1	13
7082.8194	7082.8228	-0.0034	14	0	14	13	0	13
7112.1489	7112.1522	-0.0033	12	6	7	11	6	6
7155.1059	7155.1066	-0.0007	12	5	7	11	5	6
7263.5446	7263.5580	-0.0134	12	7	6	11	7	5
7287.9830	7287.9805	0.0025	14	1	13	13	1	12
7287.9830	7287.9805	0.0025	14	2	13	13	2	12
7315.0671	7315.0578	0.0093	12	9	3	11	9	2
7409.4244	7409.4239	0.0005	13	5	9	12	5	8
7410.3720	7410.3708	0.0012	13	4	9	12	4	8
7440.4575	7440.4594	-0.0019	12	6	6	11	6	5
7446.1096	7446.1106	-0.0010	12	8	4	11	8	3
7493.3855	7493.3822	0.0033	14	3	12	13	3	11
7493.3855	7493.3824	0.0031	14	2	12	13	2	11
7581.3650	7581.3659	-0.0009	15	0	15	14	0	14
7581.3650	7581.3659	-0.0009	15	1	15	14	1	14
7617.8919	7617.8926	-0.0007	13	6	8	12	6	7
7631.3461	7631.3387	0.0074	13	5	8	12	5	7
7699.4537	7699.4459	0.0078	14	4	11	13	4	10
7699.4537	7699.4530	0.0007	14	3	11	13	3	10

7786.5133	7786.5118	0.0015	15	2	14	14	2	13
7786.5133	7786.5118	0.0015	15	1	14	14	1	13
7805.6601	7805.6594	0.0007	13	7	7	12	7	6
7894.7797	7894.7863	-0.0066	13	10	4	12	10	3
7901.5190	7901.5189	0.0001	13	6	7	12	6	6
7907.0020	7907.0070	-0.0050	14	5	10	13	5	9
7907.2027	7907.2081	-0.0054	14	4	10	13	4	9
7919.0460	7919.0461	-0.0001	13	8	6	12	8	5
7933.0805	7933.0767	0.0038	13	9	5	12	9	4
7991.8564	7991.8507	0.0057	15	2	13	14	2	12
7991.8564	7991.8507	0.0057	15	3	13	14	3	12

4.2 [2]RS

Supplementary Table 5: Observed and calculated rotational transitions (MHz) for the [2]RS dimer.

Observed	Calculated	Obs-Calc	J′	K_a′	K_c′	J″	K_a″	K_c″
2235.0892	2235.0875	0.0017	3	0	3	2	1	2
2268.0090	2268.0075	0.0015	3	1	3	2	1	2
2337.5898	2337.5867	0.0031	3	2	1	2	2	0
2490.8267	2490.8222	0.0045	3	1	2	2	0	2
2574.1314	2574.1311	0.0003	3	2	1	2	1	1
2628.3381	2628.3380	0.0001	3	2	2	2	1	2
2764.9093	2764.9132	-0.0039	3	3	1	2	2	0
2765.8589	2765.8589	0.0000	3	3	0	2	2	0
2771.3318	2771.3356	-0.0038	3	3	1	2	2	1
2772.2796	2772.2812	-0.0016	3	3	0	2	2	1
2902.7099	2902.7089	0.0010	4	1	3	3	2	1
2932.8633	2932.8813	-0.0180	4	1	3	3	2	2
3001.5175	3001.5214	-0.0039	4	0	4	3	1	3
3018.3622	3018.3623	-0.0001	4	1	4	3	1	3
3034.4380	3034.4415	-0.0035	4	0	4	3	0	3
3051.2832	3051.2824	0.0008	4	1	4	3	0	3
3080.0494	3080.0508	-0.0014	4	2	3	3	2	2

3101.0638	3101.0641	-0.0003	4	3	1	3	3	0
3125.3995	3125.3996	-0.0001	4	1	3	3	1	2
3130.6108	3130.6058	0.0050	4	2	2	3	2	1
3272.5680	3272.5691	-0.0011	4	2	3	3	1	2
3326.1340	3326.1318	0.0022	4	1	3	3	0	3
3353.2979	3353.2965	0.0014	4	2	2	3	1	2
3440.3826	3440.3812	0.0014	4	2	3	3	1	3
3483.3328	3483.3272	0.0056	5	0	5	4	1	3
3522.9153	3522.9245	-0.0092	4	3	2	3	2	1
3523.8951	3523.9033	-0.0082	5	2	3	4	3	1
3529.3329	3529.3363	-0.0034	4	3	1	3	2	1
3553.0943	3553.0969	-0.0026	4	3	2	3	2	2
3660.9861	3660.9875	-0.0014	5	1	4	4	2	2
3720.9756	3720.9781	-0.0025	4	4	1	3	3	0
3721.0926	3721.0929	-0.0003	4	4	0	3	3	0
3721.9236	3721.9237	-0.0001	4	4	1	3	3	1
3722.0342	3722.0386	-0.0044	4	4	0	3	3	1
3741.7204	3741.7150	0.0054	5	1	4	4	2	3
3758.1809	3758.1767	0.0042	5	0	5	4	1	4
3765.8824	3765.8791	0.0033	5	1	5	4	1	4
3775.0152	3775.0175	-0.0023	5	0	5	4	0	4
3782.7156	3782.7200	-0.0044	5	1	5	4	0	4
3842.1708	3842.1733	-0.0025	5	2	4	4	2	3
3888.0130	3888.0025	0.0105	5	3	2	4	3	1
3888.8769	3888.8845	-0.0076	5	1	4	4	1	3
3922.6398	3922.6337	0.0061	5	2	3	4	2	2
3989.3383	3989.3428	-0.0045	5	2	4	4	1	3
4150.5314	4150.5307	0.0007	5	2	3	4	1	3
4180.5825	4180.5748	0.0077	5	1	4	4	0	4
4262.7119	4262.7119	-0.0000	5	3	3	4	2	2
4264.1883	4264.1922	-0.0039	5	2	4	4	1	4
4286.7323	4286.7330	-0.0007	5	3	2	4	2	2
4343.4335	4343.4394	-0.0059	5	3	3	4	2	3
4343.6182	4343.6179	0.0003	6	2	4	5	3	2
4490.4651	4490.4647	0.0004	5	4	2	4	3	1
4491.4792	4491.4773	0.0019	5	4	1	4	3	1

4496.8746	4496.8764	-0.0018	5	4	2	4	3	2
4497.8873	4497.8891	-0.0018	5	4	1	4	3	2
4508.0911	4508.0859	0.0052	6	0	6	5	1	5
4511.3592	4511.3600	-0.0008	6	1	6	5	1	5
4515.7923	4515.7884	0.0039	6	0	6	5	0	5
4519.0617	4519.0625	-0.0008	6	1	6	5	0	5
4539.3477	4539.3550	-0.0073	6	1	5	5	2	4
4599.7410	4599.7425	-0.0015	6	2	5	5	2	4
4643.1298	4643.1327	-0.0029	6	3	4	5	3	3
4648.2656	4648.2685	-0.0029	6	4	3	5	4	2
4652.1291	4652.1349	-0.0058	6	4	2	5	4	1
4674.3213	4674.3163	0.0050	5	5	0	4	4	0
4674.4151	4674.4185	-0.0034	5	5	1	4	4	1
4700.1995	4700.2008	-0.0013	6	2	5	5	1	4
4707.7171	4707.7172	-0.0001	6	2	4	5	2	3
4780.6790	4780.6733	0.0057	7	3	5	6	4	3
4808.2132	4808.2111	0.0021	7	2	6	6	3	4
4910.7830	4910.7869	-0.0039	7	3	4	6	4	2
4969.3610	4969.3634	-0.0024	6	2	4	5	1	4
4983.2098	4983.2109	-0.0011	6	3	4	5	2	3
5045.3773	5045.3706	0.0067	6	1	5	5	0	5
5047.4074	5047.4048	0.0026	6	3	3	5	2	3
5051.4676	5051.4729	-0.0053	7	1	6	6	2	4
5098.0601	5098.0556	0.0045	6	2	5	5	1	5
5142.5524	5142.5531	-0.0007	7	2	5	6	3	3
5144.4006	5144.3988	0.0018	6	3	4	5	2	4
5250.7297	5250.7306	-0.0009	6	4	3	5	3	2
5254.2892	5254.2864	0.0028	7	0	7	6	1	6
5255.6118	5255.6096	0.0022	6	4	2	5	3	2
5257.5661	5257.5605	0.0056	7	0	7	6	0	6
5258.8880	5258.8858	0.0022	7	1	7	6	0	6
5274.7494	5274.7517	-0.0023	6	4	3	5	3	3
5279.6307	5279.6307	0.0000	6	4	2	5	3	3
5320.6363	5320.6355	0.0008	7	1	6	6	2	5
5352.8678	5352.8674	0.0004	7	2	6	6	2	5
5381.0184	5381.0230	-0.0046	7	1	6	6	1	5

5412.2878	5412.2922	-0.0044	7	3	5	6	3	4
5413.2701	5413.2549	0.0152	7	2	6	6	1	5
5422.6047	5422.5943	0.0104	7	5	2	6	5	1
5426.4679	5426.4660	0.0019	7	4	4	6	4	3
5438.3876	5438.3919	-0.0043	7	4	3	6	4	2
5446.8223	5446.8227	-0.0004	6	5	2	5	4	1
5446.9633	5446.9590	0.0043	6	5	1	5	4	1
5447.8363	5447.8353	0.0010	6	5	2	5	4	2
5447.9701	5447.9716	-0.0015	6	5	1	5	4	2
5482.2348	5482.2406	-0.0058	7	2	5	6	2	4
5483.0915	5483.0910	0.0005	7	3	4	6	3	3
5627.1943	5627.1881	0.0062	6	6	0	5	5	0
5687.7855	5687.7860	-0.0005	7	3	5	6	2	4
5811.7914	5811.7907	0.0007	7	2	5	6	1	5
5822.7917	5822.7786	0.0131	7	3	4	6	2	4
5902.9054	5902.9121	-0.0067	8	2	6	7	3	4
5910.6083	5910.6051	0.0032	7	1	6	6	0	6
5939.5618	5939.5629	-0.0011	7	2	6	6	1	6
5956.9482	5956.9485	-0.0003	7	3	5	6	2	5
5993.8852	5993.8911	-0.0059	7	4	4	6	3	3
5998.6983	5998.6979	0.0004	8	0	8	7	1	7
5999.2209	5999.2161	0.0048	8	1	8	7	1	7
6000.0003	6000.0232	-0.0229	8	0	8	7	0	7
6000.5449	6000.5414	0.0035	8	1	8	7	0	7
6010.6959	6010.6961	-0.0002	7	4	3	6	3	3
6058.0837	6058.0849	-0.0012	7	4	4	6	3	4
6086.5653	6086.5529	0.0124	8	1	7	7	2	6
6102.2254	6102.2247	0.0007	8	2	7	7	2	6
6134.4620	6134.4566	0.0054	8	2	7	7	1	6
6176.7779	6176.7765	0.0014	8	3	6	7	3	5
6216.6273	6216.6217	0.0056	7	5	3	6	4	2
6217.4186	6217.4185	0.0001	7	5	2	6	4	2
6221.5042	6221.5007	0.0035	7	5	3	6	4	3
6222.3001	6222.2975	0.0026	7	5	2	6	4	3
6243.4441	6243.4500	-0.0059	8	2	6	7	2	5
6400.3006	6400.2970	0.0036	7	6	1	6	5	1

6400.4118	6400.4167	-0.0049	7	6	2	6	5	2
6580.0045	6580.0055	-0.0010	7	7	0	6	6	0
6614.0625	6614.0637	-0.0012	9	2	7	8	3	5
6620.2134	6620.2132	0.0002	8	3	5	7	2	5
6674.2146	6674.2178	-0.0032	8	2	6	7	1	6
6760.1684	6760.1693	-0.0009	8	4	4	7	3	4
6771.8300	6771.8294	0.0006	8	1	7	7	0	7
6780.8609	6780.8577	0.0032	8	3	6	7	2	6
6786.1744	6786.1759	-0.0015	8	2	7	7	1	7
6848.8191	6848.8195	-0.0004	9	2	8	8	2	7
6849.5890	6849.5962	-0.0072	8	4	5	7	3	5
6864.4873	6864.4914	-0.0041	9	2	8	8	1	7
6982.9246	6982.9007	0.0239	8	5	3	7	4	3
6996.3786	6996.3885	-0.0099	8	5	4	7	4	4
7034.6881	7034.6883	-0.0002	9	4	5	8	4	4
7066.7453	7066.7285	0.0168	9	3	6	8	3	5
7172.4959	7172.5020	-0.0061	8	6	3	7	5	2
7172.6446	7172.6158	0.0288	8	6	2	7	5	2
7173.2903	7173.2987	-0.0084	8	6	3	7	5	3
7173.4049	7173.4126	-0.0077	8	6	2	7	5	3
7353.2260	7353.2088	0.0172	8	7	1	7	6	1
7443.4921	7443.4916	0.0005	9	3	6	8	2	6
7515.1829	7515.1823	0.0006	9	4	5	8	3	5
7532.8048	7532.8096	-0.0048	8	8	0	7	7	0
7546.2530	7546.2598	-0.0068	9	2	7	8	1	7
7614.7431	7614.7390	0.0041	9	3	7	8	2	7
7651.4597	7651.4630	-0.0033	9	4	6	8	3	6
7739.7556	7739.7549	0.0007	9	5	4	8	4	4
7749.4484	7749.4530	-0.0046	10	4	7	9	4	6
7774.3827	7774.3860	-0.0033	9	5	5	8	4	5
7774.6242	7774.6239	0.0003	10	3	8	9	2	7
7942.5391	7942.5432	-0.0041	9	6	4	8	5	3
7943.0946	7943.0956	-0.0010	9	6	3	8	5	3
7945.8600	7945.8604	-0.0004	9	6	4	8	5	4

4.3 [3]RR

Supplementary Table 6: Observed and calculated rotational transitions (MHz) for the [3]RR dimer.

Observed	Calculated	Obs-Calc	J′	K_a'	K_c'	J″	K_a''	K_c''
2006.4568	2006.4591	-0.0023	3	0	3	2	0	2
2010.0319	2010.0395	-0.0076	6	6	0	6	5	2
2018.6597	2018.6585	0.0012	3	2	2	2	2	1
2030.8666	2030.8616	0.0050	3	2	1	2	2	0
2058.0693	2058.0731	-0.0038	3	1	2	2	1	1
2120.3568	2120.3587	-0.0019	3	1	3	2	0	2
2255.3022	2255.3125	-0.0103	4	1	3	3	2	1
2286.0621	2286.0599	0.0022	3	1	2	2	0	2
2330.6453	2330.6458	-0.0005	14	7	8	14	6	8
2334.7403	2334.7348	0.0055	14	7	7	14	6	9
2343.7405	2343.7378	0.0027	13	7	7	13	6	7
2345.4092	2345.4054	0.0038	13	7	6	13	6	8
2370.8000	2370.8006	-0.0006	9	7	2	9	6	3
2370.8153	2370.8158	-0.0005	9	7	3	9	6	4
2373.8044	2373.8077	-0.0033	8	7	1	8	6	2
2373.8141	2373.8108	0.0033	8	7	2	8	6	3
2375.9228	2375.9170	0.0058	7	7	1	7	6	2
2375.9228	2375.9165	0.0063	7	7	0	7	6	1
2382.9104	2382.9170	-0.0066	4	0	4	3	1	2
2527.3539	2527.3561	-0.0022	3	2	2	2	1	1
2542.6695	2542.6734	-0.0039	3	2	1	2	1	1
2548.6157	2548.6182	-0.0025	4	0	4	3	1	3
2610.3167	2610.3171	-0.0004	3	2	2	2	1	2
2625.6376	2625.6344	0.0032	3	2	1	2	1	2
2630.6148	2630.6122	0.0026	4	1	4	3	1	3
2662.5144	2662.5178	-0.0034	4	0	4	3	0	3
2689.1065	2689.1059	0.0006	4	2	3	3	2	2
2697.1495	2697.1493	0.0002	4	3	2	3	3	1
2698.4661	2698.4635	0.0026	4	3	1	3	3	0
2718.1189	2718.1159	0.0030	4	2	2	3	2	1
2729.0666	2729.0618	0.0048	12	8	4	12	7	5

2729.0778	2729.0799	-0.0021	12	8	5	12	7	6
2733.6620	2733.6597	0.0023	11	8	3	11	7	4
2733.6620	2733.6645	-0.0025	11	8	4	11	7	5
2737.1884	2737.1888	-0.0004	10	8	2	10	7	3
2737.1884	2737.1899	-0.0015	10	8	3	10	7	4
2739.8373	2739.8382	-0.0009	9	8	1	9	7	2
2739.8373	2739.8384	-0.0011	9	8	2	9	7	3
2739.9125	2739.9128	-0.0003	4	1	3	3	1	2
2741.7740	2741.7729	0.0011	8	8	0	8	7	1
2741.7740	2741.7729	0.0011	8	8	1	8	7	2
2744.5079	2744.5118	-0.0039	4	1	4	3	0	3
2934.3531	2934.3575	-0.0044	3	3	1	2	2	0
2934.5805	2934.5784	0.0021	3	3	0	2	2	0
2937.4706	2937.4717	-0.0011	3	3	1	2	2	1
2937.6865	2937.6926	-0.0061	3	3	0	2	2	1
3019.5161	3019.5135	0.0026	4	1	3	3	0	3
3091.2994	3091.3104	-0.0110	14	9	5	14	8	6
3091.3254	3091.3158	0.0096	14	9	6	14	8	7
3096.2875	3096.2902	-0.0027	13	9	4	13	8	5
3096.2875	3096.2917	-0.0042	13	9	5	13	8	6
3100.2678	3100.2672	0.0006	12	9	3	12	8	4
3100.2678	3100.2676	0.0002	12	9	4	12	8	5
3103.3891	3103.3935	-0.0044	11	9	2	11	8	3
3103.3891	3103.3935	-0.0044	11	9	3	11	8	4
3105.8028	3105.8047	-0.0019	10	9	1	10	8	2
3105.8028	3105.8047	-0.0019	10	9	2	10	8	3
3107.6220	3107.6224	-0.0004	9	9	1	9	8	2
3107.6220	3107.6224	-0.0004	9	9	0	9	8	1
3202.7139	3202.7162	-0.0023	4	2	2	3	1	2
3229.3374	3229.3311	0.0063	5	0	5	4	1	4
3283.7558	3283.7569	-0.0011	5	1	5	4	1	4
3311.3229	3311.3251	-0.0022	5	0	5	4	0	4
3324.0914	3324.0901	0.0013	4	2	3	3	1	3
3357.4912	3357.4923	-0.0011	5	2	4	4	2	3
3365.7469	3365.7509	-0.0040	5	1	5	4	0	4
3372.9679	3372.9802	-0.0123	5	3	3	4	3	2

3377.4940	3377.4975	-0.0035	5	3	2	4	3	1
3410.4780	3410.4754	0.0026	5	2	3	4	2	2
3417.5048	3417.5074	-0.0026	5	1	4	4	1	3
3447.0479	3447.0501	-0.0022	17	10	8	17	9	9
3447.0479	3447.0451	0.0028	17	10	7	17	9	8
3453.4417	3453.4351	0.0066	16	10	6	16	9	7
3453.4417	3453.4366	0.0051	16	10	7	16	9	8
3458.7418	3458.7432	-0.0014	15	10	6	15	9	7
3458.7418	3458.7428	-0.0010	15	10	5	15	9	6
3463.1069	3463.1074	-0.0005	14	10	5	14	9	6
3463.1069	3463.1073	-0.0004	14	10	4	14	9	5
3466.6524	3466.6543	-0.0019	13	10	4	13	9	5
3466.6524	3466.6542	-0.0018	13	10	3	13	9	4
3469.4981	3469.4973	0.0008	12	10	3	12	9	4
3469.4981	3469.4973	0.0008	12	10	2	12	9	3
3471.7390	3471.7397	-0.0007	11	10	2	11	9	3
3471.7390	3471.7397	-0.0007	11	10	1	11	9	2
3473.4753	3473.4751	0.0002	10	10	0	10	9	1
3473.4753	3473.4751	0.0002	10	10	1	10	9	2
3490.2656	3490.2658	-0.0002	7	3	4	6	4	2
3492.3217	3492.3204	0.0013	6	0	6	5	1	4
3600.6411	3600.6452	-0.0041	4	3	2	3	2	1
3602.1800	3602.1802	-0.0002	4	3	1	3	2	1
3615.9586	3615.9625	-0.0039	4	3	2	3	2	2
3617.4990	3617.4976	0.0014	4	3	1	3	2	2
3633.3302	3633.3314	-0.0012	6	1	5	5	2	3
3718.5250	3718.5293	-0.0043	7	2	6	6	3	4
3728.3675	3728.3467	0.0208	7	1	7	6	2	5
3730.6439	3730.6418	0.0021	6	1	5	5	2	4
3765.5318	3765.5249	0.0069	8	4	5	7	5	3
3774.5085	3774.5032	0.0053	5	1	4	4	0	4
3775.9631	3775.9685	-0.0054	5	2	4	4	1	3
3825.7555	3825.7540	0.0015	16	11	5	16	10	6
3825.7555	3825.7540	0.0015	16	11	5	16	10	7
3825.7555	3825.7540	0.0015	16	11	6	16	10	6
3825.7555	3825.7540	0.0015	16	11	6	16	10	7

3829.6704	3829.6740	-0.0036	15	11	4	15	10	5
3829.6704	3829.6740	-0.0036	15	11	5	15	10	6
3829.6704	3829.6740	-0.0036	15	11	5	15	10	5
3829.6704	3829.6740	-0.0036	15	11	4	15	10	6
3832.9067	3832.9074	-0.0007	14	11	4	14	10	5
3832.9067	3832.9074	-0.0007	14	11	4	14	10	4
3832.9067	3832.9074	-0.0007	14	11	3	14	10	5
3832.9067	3832.9074	-0.0007	14	11	3	14	10	4
3835.5419	3835.5428	-0.0009	13	11	2	13	10	3
3835.5419	3835.5428	-0.0009	13	11	2	13	10	4
3835.5419	3835.5428	-0.0009	13	11	3	13	10	3
3835.5419	3835.5428	-0.0009	13	11	3	13	10	4
3837.6616	3837.6614	0.0002	12	11	1	12	10	2
3837.6616	3837.6614	0.0002	12	11	2	12	10	3
3837.6616	3837.6614	0.0002	12	11	2	12	10	2
3837.6616	3837.6614	0.0002	12	11	1	12	10	3
3839.3365	3839.3377	-0.0012	11	11	0	11	10	1
3839.3365	3839.3377	-0.0012	11	11	0	11	10	2
3839.3365	3839.3377	-0.0012	11	11	1	11	10	1
3839.3365	3839.3377	-0.0012	11	11	1	11	10	2
3873.2809	3873.2788	0.0021	5	2	3	4	1	3
3901.0724	3901.0726	-0.0002	6	0	6	5	1	5
3934.8321	3934.8305	0.0016	6	1	6	5	1	5
3955.5025	3955.4985	0.0040	6	0	6	5	0	5
3975.6665	3975.6601	0.0064	4	4	0	3	3	0
3975.8678	3975.8680	-0.0002	4	4	1	3	3	1
3989.2534	3989.2564	-0.0030	6	1	6	5	0	5
3990.0826	3990.0841	-0.0015	7	2	5	6	3	3
4023.3726	4023.3745	-0.0019	6	2	5	5	2	4
4045.0563	4045.0545	0.0018	6	5	2	5	5	1
4045.0563	4045.0614	-0.0051	6	5	1	5	5	0
4047.6894	4047.6882	0.0012	6	4	3	5	4	2
4048.1505	4048.1484	0.0021	6	4	2	5	4	1
4048.9158	4048.9062	0.0096	6	3	4	5	3	3
4050.9666	4050.9702	-0.0036	5	2	4	4	1	4
4060.5553	4060.5492	0.0061	6	3	3	5	3	2

4089.1009	4089.1028	-0.0019	6	1	5	5	1	4
4104.6917	4104.6884	0.0033	6	2	4	5	2	3
4148.2864	4148.2806	0.0058	5	2	3	4	1	4
4183.2339	4183.2358	-0.0019	19	12	7	19	11	9
4183.2339	4183.2358	-0.0019	19	12	8	19	11	8
4183.2339	4183.2358	-0.0019	19	12	8	19	11	9
4183.2339	4183.2358	-0.0019	19	12	7	19	11	8
4188.2397	4188.2390	0.0007	18	12	6	18	11	7
4188.2397	4188.2390	0.0007	18	12	7	18	11	8
4188.2397	4188.2390	0.0007	18	12	7	18	11	7
4188.2397	4188.2390	0.0007	18	12	6	18	11	8
4192.4941	4192.4925	0.0016	17	12	6	17	11	6
4192.4941	4192.4925	0.0016	17	12	5	17	11	7
4192.4941	4192.4925	0.0016	17	12	5	17	11	6
4192.4941	4192.4925	0.0016	17	12	6	17	11	7
4196.0786	4196.0794	-0.0008	16	12	4	16	11	5
4196.0786	4196.0794	-0.0008	16	12	5	16	11	5
4196.0786	4196.0794	-0.0008	16	12	5	16	11	6
4196.0786	4196.0794	-0.0008	16	12	4	16	11	6
4199.0773	4199.0767	0.0006	15	12	3	15	11	4
4199.0773	4199.0767	0.0006	15	12	4	15	11	4
4199.0773	4199.0767	0.0006	15	12	4	15	11	5
4199.0773	4199.0767	0.0006	15	12	3	15	11	5
4201.5513	4201.5554	-0.0041	14	12	3	14	11	4
4201.5513	4201.5554	-0.0041	14	12	3	14	11	3
4201.5513	4201.5554	-0.0041	14	12	2	14	11	4
4201.5513	4201.5554	-0.0041	14	12	2	14	11	3
4203.5777	4203.5812	-0.0035	13	12	2	13	11	2
4203.5777	4203.5812	-0.0035	13	12	1	13	11	3
4203.5777	4203.5812	-0.0035	13	12	2	13	11	3
4203.5777	4203.5812	-0.0035	13	12	1	13	11	2
4205.2105	4205.2147	-0.0042	12	12	0	12	11	1
4205.2105	4205.2147	-0.0042	12	12	1	12	11	2
4205.2105	4205.2147	-0.0042	12	12	1	12	11	1
4205.2105	4205.2147	-0.0042	12	12	0	12	11	2
4255.5073	4255.5096	-0.0023	5	3	3	4	2	2

4261.5578	4261.5618	-0.0040	5	3	2	4	2	2
4273.8917	4273.8978	-0.0061	8	1	8	7	2	6
4281.5811	4281.5829	-0.0018	7	1	6	6	2	4
4299.8337	4299.8368	-0.0031	5	3	3	4	2	3
4305.8910	4305.8891	0.0019	5	3	2	4	2	3
4312.1256	4312.1253	0.0003	11	8	4	10	9	1
4312.1256	4312.1253	0.0003	11	8	4	10	9	2
4312.1256	4312.1254	0.0002	11	8	3	10	9	2
4312.1256	4312.1254	0.0002	11	8	3	10	9	1
4340.6432	4340.6502	-0.0070	8	2	7	7	3	5
4381.8347	4381.8356	-0.0009	6	2	5	5	1	4
4449.2244	4449.2209	0.0035	9	4	6	8	5	4
4464.4906	4464.4923	-0.0017	9	4	5	8	5	3
4488.8329	4488.8391	-0.0062	8	0	8	7	1	6
4552.2698	4552.2809	-0.0111	6	1	5	5	0	5
4555.1414	4555.1389	0.0025	19	13	7	19	12	8
4555.1414	4555.1389	0.0025	19	13	6	19	12	8
4555.1414	4555.1389	0.0025	19	13	6	19	12	7
4555.1414	4555.1389	0.0025	19	13	7	19	12	7
4559.0500	4559.0474	0.0026	18	13	5	18	12	6
4559.0500	4559.0474	0.0026	18	13	6	18	12	7
4559.0500	4559.0474	0.0026	18	13	6	18	12	6
4559.0500	4559.0474	0.0026	18	13	5	18	12	7
4560.4589	4560.4599	-0.0010	6	2	4	5	1	4
4564.2282	4564.2293	-0.0011	7	0	7	6	1	6
4567.5510	4567.5517	-0.0007	15	13	3	15	12	4
4567.5510	4567.5517	-0.0007	15	13	3	15	12	3
4567.5510	4567.5517	-0.0007	15	13	2	15	12	4
4567.5510	4567.5517	-0.0007	15	13	2	15	12	3
4569.5092	4569.5071	0.0021	14	13	1	14	12	3
4569.5092	4569.5071	0.0021	14	13	2	14	12	2
4569.5092	4569.5071	0.0021	14	13	2	14	12	3
4569.5092	4569.5071	0.0021	14	13	1	14	12	2
4571.1178	4571.1099	0.0079	13	13	0	13	12	2
4571.1178	4571.1099	0.0079	13	13	1	13	12	1
4571.1178	4571.1099	0.0079	13	13	1	13	12	2

4571.1178	4571.1099	0.0079	13	13	0	13	12	1
4584.1007	4584.1040	-0.0033	7	1	7	6	1	6
4597.9878	4597.9872	0.0006	7	0	7	6	0	6
4617.8633	4617.8619	0.0014	7	1	7	6	0	6
4648.4824	4648.4830	-0.0006	5	4	2	4	3	1
4648.5928	4648.5992	-0.0064	5	4	1	4	3	1
4650.0163	4650.0180	-0.0017	5	4	2	4	3	2
4650.1357	4650.1342	0.0015	5	4	1	4	3	2
4684.2684	4684.2704	-0.0020	11	7	4	10	8	3
4684.2684	4684.2655	0.0029	11	7	5	10	8	3
4684.2684	4684.2654	0.0030	11	7	5	10	8	2
4684.2684	4684.2704	-0.0020	11	7	4	10	8	2
4686.4008	4686.4056	-0.0048	7	2	6	6	2	5
4718.8116	4718.8146	-0.0030	7	6	1	6	6	0
4718.8116	4718.8142	-0.0026	7	6	2	6	6	1
4724.2650	4724.2645	0.0005	7	3	5	6	3	4
4725.0199	4725.0247	-0.0048	7	4	4	6	4	3
4725.8350	4725.8398	-0.0048	8	2	6	7	3	4
4726.5246	4726.5338	-0.0092	7	4	3	6	4	2
4748.9798	4748.9667	0.0131	7	3	4	6	3	3
4752.9381	4752.9400	-0.0019	7	1	6	6	1	5
4790.5923	4790.5878	0.0045	6	2	5	5	1	5
4797.0370	4797.0312	0.0058	7	2	5	6	2	4
4806.2893	4806.2906	-0.0013	9	1	9	8	2	7
4892.5940	4892.5924	0.0016	8	1	7	7	2	5
4893.9383	4893.9404	-0.0021	6	3	4	5	2	3
4911.6326	4911.6355	-0.0029	6	3	3	5	2	3
4942.0429	4942.0484	-0.0055	9	3	6	8	4	4
4945.6457	4945.6568	-0.0111	9	2	8	8	3	6
4964.6434	4964.6387	0.0047	9	0	9	8	1	7
4979.1332	4979.1383	-0.0051	7	2	6	6	1	5
4991.2504	4991.2507	-0.0003	6	3	4	5	2	4
5008.9545	5008.9459	0.0086	6	3	3	5	2	4
5015.4470	5015.4465	0.0005	5	5	1	4	4	1
5220.7003	5220.6996	0.0007	8	0	8	7	1	7
5231.9569	5231.9567	0.0002	8	1	8	7	1	7

5240.5723	5240.5742	-0.0019	8	0	8	7	0	7
5251.8335	5251.8314	0.0021	8	1	8	7	0	7
5268.3882	5268.3882	-0.0000	7	2	5	6	1	5
5318.6699	5318.6737	-0.0038	6	4	3	5	3	2
5319.2481	5319.2501	-0.0020	6	4	2	5	3	2
5324.7259	5324.7259	-0.0000	6	4	3	5	3	3
5325.3006	5325.3024	-0.0018	6	4	2	5	3	3
5327.8951	5327.8915	0.0036	10	1	10	9	2	8
5346.3828	5346.3853	-0.0025	8	2	7	7	2	6
5349.7322	5349.7224	0.0098	7	1	6	6	0	6
5392.5838	5392.5808	0.0030	8	7	2	7	7	1
5392.5838	5392.5808	0.0030	8	7	1	7	7	0
5394.6875	5394.6869	0.0006	8	6	3	7	6	2
5394.7000	5394.6896	0.0104	8	6	2	7	6	1
5398.1785	5398.1780	0.0005	8	5	4	7	5	3
5398.2911	5398.2942	-0.0031	8	3	6	7	3	5
5403.1874	5403.1894	-0.0020	8	4	5	7	4	4
5407.2290	5407.2340	-0.0050	8	4	4	7	4	3
5408.0478	5408.0407	0.0071	8	1	7	7	1	6
5437.2208	5437.2233	-0.0025	10	0	10	9	1	8
5443.2891	5443.2912	-0.0021	8	3	5	7	3	4
5446.3932	5446.3973	-0.0041	10	3	8	9	4	6
5448.4460	5448.4415	0.0045	9	2	7	8	3	5
5463.0479	5463.0471	0.0008	9	1	8	8	2	6
5484.7195	5484.7224	-0.0029	8	2	6	7	2	5
5513.5088	5513.5164	-0.0076	7	3	5	6	2	4
5532.7429	5532.7444	-0.0015	10	2	9	9	3	7
5535.8320	5535.8360	-0.0040	9	2	7	8	3	6
5542.1612	5542.1628	-0.0016	7	2	6	6	1	6
5555.9132	5555.9138	-0.0006	7	3	4	6	2	4
5572.5699	5572.5837	-0.0138	8	2	7	7	1	6
5658.0994	5658.0991	0.0003	21	16	6	21	15	7
5658.0994	5658.0991	0.0003	21	16	5	21	15	7
5658.0994	5658.0991	0.0003	21	16	5	21	15	6
5658.0994	5658.0991	0.0003	21	16	6	21	15	6
5660.9387	5660.9404	-0.0017	20	16	5	20	15	6

5660.9387	5660.9404	-0.0017	20	16	5	20	15	5
5660.9387	5660.9404	-0.0017	20	16	4	20	15	6
5660.9387	5660.9404	-0.0017	20	16	4	20	15	5
5663.4068	5663.4102	-0.0034	19	16	4	19	15	5
5663.4068	5663.4102	-0.0034	19	16	4	19	15	4
5663.4068	5663.4102	-0.0034	19	16	3	19	15	5
5663.4068	5663.4102	-0.0034	19	16	3	19	15	4
5665.5392	5665.5441	-0.0049	18	16	2	18	15	3
5665.5392	5665.5441	-0.0049	18	16	3	18	15	4
5665.5392	5665.5441	-0.0049	18	16	3	18	15	3
5665.5392	5665.5441	-0.0049	18	16	2	18	15	4
5667.3833	5667.3753	0.0080	17	16	1	17	15	3
5667.3833	5667.3753	0.0080	17	16	2	17	15	3
5667.3833	5667.3753	0.0080	17	16	1	17	15	2
5667.3833	5667.3753	0.0080	17	16	2	17	15	2
5671.3907	5671.3944	-0.0037	13	8	6	12	9	3
5671.3907	5671.3944	-0.0037	13	8	6	12	9	4
5671.3907	5671.3959	-0.0052	13	8	5	12	9	4
5671.3907	5671.3959	-0.0052	13	8	5	12	9	3
5689.1069	5689.0930	0.0139	6	5	1	5	4	1
5689.1927	5689.2017	-0.0090	6	5	2	5	4	2
5692.1423	5692.1407	0.0016	7	3	5	6	2	5
5734.5559	5734.5382	0.0177	7	3	4	6	2	5
5815.1572	5815.1559	0.0013	11	4	8	10	5	5
5817.1277	5817.1257	0.0020	11	4	8	10	5	6
5841.1175	5841.1315	-0.0140	11	1	11	10	2	9
5872.5844	5872.5832	0.0012	9	0	9	8	1	8
5878.7837	5878.7781	0.0056	9	1	9	8	1	8
5883.8415	5883.8403	0.0012	9	0	9	8	0	8
5884.7942	5884.7893	0.0049	11	4	7	10	5	5
5890.0387	5890.0353	0.0034	9	1	9	8	0	8
5890.6511	5890.6341	0.0170	9	1	8	8	2	7
5983.1514	5983.1492	0.0022	7	4	4	6	3	3
5985.2329	5985.2348	-0.0019	7	4	3	6	3	3
6000.1674	6000.1707	-0.0033	8	2	6	7	1	6
6000.8421	6000.8444	-0.0023	7	4	4	6	3	4

6002.9323	6002.9300	0.0023	7	4	3	6	3	4
6003.3025	6003.3007	0.0018	9	2	8	8	2	7
6027.2713	6027.2709	0.0004	7	3	4	6	1	5
6055.0915	6055.0940	-0.0025	6	6	1	5	5	1
6055.1665	6055.1771	-0.0106	9	1	8	8	1	7
6066.3407	6066.3381	0.0026	9	8	2	8	8	1
6066.3407	6066.3381	0.0026	9	8	1	8	8	0
6068.2710	6068.2726	-0.0016	9	7	3	8	7	2
6068.2710	6068.2728	-0.0018	9	7	2	8	7	1
6070.2501	6070.2504	-0.0003	9	3	7	8	3	6
6071.2708	6071.2799	-0.0091	9	6	3	8	6	2
6076.1382	6076.1477	-0.0095	9	5	5	8	5	4
6076.6192	6076.6227	-0.0035	9	5	4	8	5	3
6081.8754	6081.8740	0.0014	9	4	6	8	4	5
6091.2089	6091.2087	0.0002	9	4	5	8	4	4
6091.3860	6091.3911	-0.0051	11	3	9	10	4	7
6102.1082	6102.1028	0.0054	11	2	10	10	3	8
6114.7772	6114.7794	-0.0022	8	3	6	7	2	5
6130.7744	6130.7776	-0.0032	12	5	8	11	6	5
6130.9844	6130.9825	0.0019	12	5	8	11	6	6
6142.2802	6142.2594	0.0208	9	3	6	8	3	5
6142.8302	6142.8272	0.0030	12	5	7	11	6	5
6145.2115	6145.2109	0.0006	10	2	8	9	3	6
6159.7827	6159.7758	0.0069	8	1	7	7	0	7
6165.8930	6165.8929	0.0001	9	2	7	8	2	6
6167.8503	6167.8437	0.0066	9	2	8	8	1	7
6202.1831	6202.1738	0.0093	8	3	5	7	2	5
6304.4458	6304.4442	0.0016	8	2	7	7	1	7
6304.6139	6304.6143	-0.0004	10	2	8	9	3	7
6362.1234	6362.1128	0.0106	7	5	3	6	4	2
6362.1546	6362.1578	-0.0032	7	5	2	6	4	2
6362.6889	6362.6892	-0.0003	7	5	3	6	4	3
6362.7301	6362.7343	-0.0042	7	5	2	6	4	3
6404.0278	6404.0293	-0.0015	8	3	6	7	2	6
6432.1732	6432.1630	0.0102	13	6	8	12	7	6
6433.7463	6433.7512	-0.0049	13	6	7	12	7	5

6454.3275	6454.3260	0.0015	11	3	8	10	4	6
6491.4410	6491.4237	0.0173	8	3	5	7	2	6
6491.7915	6491.7881	0.0034	11	1	10	10	2	8
6497.2860	6497.2907	-0.0047	12	4	9	11	5	7
6521.5737	6521.5668	0.0069	10	0	10	9	1	9
6524.9096	6524.9016	0.0080	10	1	10	9	1	9
6527.7660	6527.7617	0.0043	10	0	10	9	0	9
6531.1025	6531.0966	0.0059	10	1	10	9	0	9
6584.3303	6584.3188	0.0115	10	1	9	9	2	8
6620.1923	6620.1913	0.0010	12	4	8	11	5	6
6624.0574	6624.0533	0.0041	7	3	4	6	0	6
6625.3119	6625.3176	-0.0057	12	4	8	11	5	7
6637.3740	6637.3719	0.0021	8	4	5	7	3	4
6643.5032	6643.5021	0.0011	8	4	4	7	3	4
6654.8625	6654.8518	0.0107	12	2	11	11	3	9
6657.3414	6657.3381	0.0033	10	2	9	9	2	8
6679.7679	6679.7694	-0.0015	8	4	5	7	3	5
6685.8992	6685.8995	-0.0003	8	4	4	7	3	5
6696.9872	6696.9854	0.0018	10	1	9	9	1	8
6700.3147	6700.3074	0.0073	9	3	7	8	2	6
6720.9751	6720.9826	-0.0075	12	3	10	11	4	8
6728.8464	6728.8536	-0.0072	7	6	2	6	5	2
6739.4959	6739.4961	-0.0002	10	3	8	9	3	7
6740.0788	6740.0749	0.0039	10	9	2	9	9	1
6740.0788	6740.0749	0.0039	10	9	1	9	9	0
6741.8962	6741.8926	0.0036	10	8	2	9	8	1
6744.5426	6744.5420	0.0006	10	7	3	9	7	2
6748.6423	6748.6475	-0.0052	10	6	5	9	6	4
6748.6932	6748.6931	0.0001	10	6	4	9	6	3
6755.1169	6755.1177	-0.0008	10	5	6	9	5	5
6756.4195	6756.4191	0.0004	10	5	5	9	5	4
6758.0270	6758.0229	0.0041	9	2	7	8	1	7
6760.5687	6760.5697	-0.0010	10	4	7	9	4	6
6770.0024	6770.0047	-0.0023	10	2	9	9	1	8
6779.6545	6779.6548	-0.0003	10	4	6	9	4	5
6805.4041	6805.4103	-0.0062	11	2	9	10	3	7

6839.0242	6839.0287	-0.0045	10	2	8	9	2	7
6842.6480	6842.6468	0.0012	10	3	7	9	3	6
6859.7099	6859.7107	-0.0008	9	3	6	8	2	6
6965.4882	6965.4833	0.0049	12	1	11	11	2	9
6974.3873	6974.3787	0.0086	9	1	8	8	0	8
7033.7491	7033.7569	-0.0078	8	5	4	7	4	3
7033.9490	7033.9504	-0.0014	8	5	3	7	4	3
7035.8407	7035.8425	-0.0018	8	5	4	7	4	4
7036.0341	7036.0360	-0.0019	8	5	3	7	4	4
7067.9645	7067.9644	0.0001	11	2	9	10	3	8
7075.7954	7075.7882	0.0072	9	2	8	8	1	8
7094.7285	7094.7312	-0.0027	7	7	0	6	6	0
7127.8957	7127.8944	0.0013	9	3	7	8	2	7
7168.8167	7168.8137	0.0030	11	0	11	10	1	10
7170.5817	7170.5780	0.0037	11	1	11	10	1	10
7171.1748	7171.1781	-0.0033	13	4	10	12	5	8
7172.1506	7172.1485	0.0021	11	0	11	10	0	10
7173.9224	7173.9129	0.0095	11	1	11	10	0	10
7192.8612	7192.8602	0.0010	13	2	12	12	3	10
7205.1100	7205.1036	0.0064	13	3	11	12	4	8
7213.1704	7213.1782	-0.0078	12	3	9	11	4	7
7263.6577	7263.6580	-0.0003	11	1	10	10	2	9
7273.9109	7273.9105	0.0004	10	3	8	9	2	7
7275.9591	7275.9547	0.0044	9	4	6	8	3	5
7282.8075	7282.8117	-0.0042	12	3	9	11	4	8
7291.4179	7291.4196	-0.0017	9	4	5	8	3	5
7308.8569	7308.8545	0.0024	11	2	10	10	2	9
7333.1297	7333.1305	-0.0008	13	3	11	12	4	9
7336.6835	7336.6773	0.0062	11	1	10	10	1	9
7341.0120	7341.0144	-0.0024	16	9	7	15	10	6
7341.0120	7341.0128	-0.0008	16	9	8	15	10	6
7341.0120	7341.0144	-0.0024	16	9	7	15	10	5
7341.0120	7341.0128	-0.0008	16	9	8	15	10	5
7363.3396	7363.3492	-0.0096	9	4	6	8	3	6
7375.5242	7375.5291	-0.0049	13	4	9	12	5	7
7378.8125	7378.8141	-0.0016	9	4	5	8	3	6

7381.8730	7381.8738	-0.0008	11	2	10	10	1	9
7387.5786	7387.5787	-0.0001	13	4	9	12	5	8
7402.3125	7402.3229	-0.0104	8	6	2	7	5	2
7402.3652	7402.3648	0.0004	8	6	3	7	5	3
7405.5678	7405.5635	0.0043	11	3	9	10	3	8
7415.5222	7415.5188	0.0034	11	9	2	10	9	1
7417.9361	7417.9300	0.0061	11	8	3	10	8	2
7421.4552	7421.4553	-0.0001	11	7	5	10	7	4
7421.7336	7421.7437	-0.0101	12	2	10	11	3	8
7426.9065	7426.9130	-0.0065	11	6	6	10	6	5
7427.0535	7427.0569	-0.0034	11	6	5	10	6	4
7435.0341	7435.0312	0.0029	11	5	7	10	5	6
7438.1849	7438.1877	-0.0028	11	5	6	10	5	5
7438.6025	7438.6005	0.0020	11	4	8	10	4	7
7473.6759	7473.6839	-0.0080	11	4	7	10	4	6
7502.8464	7502.8462	0.0002	11	2	9	10	2	8
7536.4562	7536.4646	-0.0084	10	3	7	9	2	7
7540.4820	7540.4943	-0.0123	11	3	8	10	3	7
7541.8610	7541.8746	-0.0136	10	2	8	9	1	8
7702.6661	7702.6705	-0.0044	9	5	5	8	4	4
7703.3562	7703.3390	0.0172	9	5	4	8	4	4
7708.8004	7708.8007	-0.0003	9	5	5	8	4	5
7709.4784	7709.4692	0.0092	9	5	4	8	4	5
7768.4943	7768.4977	-0.0034	8	7	2	7	6	2
7787.5391	7787.5238	0.0153	10	1	9	9	0	9
7815.0648	7815.0578	0.0070	12	0	12	11	1	11
7815.9889	7815.9782	0.0107	12	1	12	11	1	11
7816.8320	7816.8222	0.0098	12	0	12	11	0	11
7817.7513	7817.7426	0.0087	12	1	12	11	0	11
7819.2199	7819.2286	-0.0087	12	2	10	11	3	9
7823.7625	7823.7676	-0.0051	15	6	9	14	7	7
7835.5109	7835.5039	0.0070	14	4	11	13	5	9
7840.4382	7840.4453	-0.0071	11	3	9	10	2	8
7854.3450	7854.3482	-0.0032	10	2	9	9	1	9
7864.0850	7864.0897	-0.0047	10	3	8	9	2	8
7883.8893	7883.8935	-0.0042	14	1	13	13	2	11

7894.2595	7894.2651	-0.0056	10	4	7	9	3	6
7917.8308	7917.8330	-0.0022	8	4	4	7	1	6
7928.8065	7928.8151	-0.0086	10	4	6	9	3	6
7956.1512	7956.1514	-0.0002	13	3	10	12	4	8
7958.3148	7958.3125	0.0023	12	2	11	11	2	10
7976.5467	7976.5414	0.0053	12	1	11	11	1	10
7991.2533	7991.2461	0.0072	13	2	11	12	3	9

4.4 [7]RR

Supplementary Table 7: Observed and calculated rotational transitions (MHz) for the [7]RR dimer.

Observed	Calculated	Obs-Calc	J′	K_a'	K_c'	J″	K_a''	K_c''
2509.6208	2509.6219	-0.0011	3	2	1	2	1	1
2517.3897	2517.3882	0.0015	4	1	4	3	1	3
2548.8190	2548.8193	-0.0003	4	0	4	3	0	3
2619.2879	2619.2882	-0.0003	3	2	2	2	1	2
2736.9697	2736.9712	-0.0015	4	1	3	3	1	2
2887.0293	2887.0310	-0.0017	3	3	1	2	2	0
2889.1243	2889.1235	0.0008	3	3	0	2	2	0
2900.7244	2900.7230	0.0014	3	3	1	2	2	1
3131.9452	3131.9471	-0.0019	5	1	5	4	1	4
3149.3372	3149.3361	0.0011	5	0	5	4	0	4
3192.0144	3192.0135	0.0009	4	2	2	3	1	2
3536.8957	3536.8980	-0.0023	4	3	1	3	2	1
3586.8115	3586.8136	-0.0021	4	3	2	3	2	2
3736.5675	3736.5656	0.0019	6	0	6	5	1	5
3742.4033	3742.4014	0.0019	6	1	6	5	1	5
3750.6123	3750.6125	-0.0002	6	0	6	5	0	5
3911.9619	3911.9624	-0.0005	5	2	3	4	1	3
3918.3552	3918.3519	0.0033	4	4	1	3	3	0
3918.6178	3918.6157	0.0021	4	4	0	3	3	0
3920.4437	3920.4443	-0.0006	4	4	1	3	3	1
3920.7114	3920.7081	0.0033	4	4	0	3	3	1

3923.8035	3923.8075	-0.0040	6	2	5	5	2	4
4002.5410	4002.5420	-0.0010	6	1	5	5	1	4
4035.8506	4035.8558	-0.0052	6	4	2	5	4	1
4147.5017	4147.4964	0.0053	6	2	4	5	2	3
4173.1950	4173.1950	0.0000	5	3	2	4	2	2
4290.8787	4290.8803	-0.0016	5	3	3	4	2	3
4348.1326	4348.1346	-0.0020	7	0	7	6	1	6
4350.4416	4350.4442	-0.0026	7	1	7	6	1	6
4353.9683	4353.9704	-0.0021	7	0	7	6	0	6
4356.2783	4356.2800	-0.0017	7	1	7	6	0	6
4580.5093	4580.5033	0.0060	5	4	1	4	3	1
4592.3278	4592.3335	-0.0057	5	4	2	4	3	2
4594.6438	4594.6550	-0.0112	5	4	1	4	3	2
4603.1012	4603.0978	0.0034	7	1	6	6	1	5
4673.7619	4673.7641	-0.0022	7	3	5	6	3	4
4676.5787	4676.5817	-0.0030	6	2	4	5	1	4
4696.2880	4696.2844	0.0036	7	5	3	6	5	2
4697.8559	4697.8526	0.0033	7	5	2	6	5	1
4705.2582	4705.2571	0.0011	7	4	4	6	4	3
4815.2769	4815.2774	-0.0005	7	2	5	6	2	4
4818.4373	4818.4386	-0.0013	6	3	3	5	2	3
4824.9000	4824.9042	-0.0042	7	3	4	6	3	3
4930.4521	4930.4531	-0.0010	6	2	5	5	1	5
4943.9314	4943.9269	0.0045	5	5	0	4	4	0
4944.1616	4944.1606	0.0010	5	5	1	4	4	1
4956.3742	4956.3740	0.0002	8	0	8	7	1	7
4957.2627	4957.2572	0.0055	8	1	8	7	1	7
4958.6808	4958.6836	-0.0028	8	0	8	7	0	7
4959.5616	4959.5668	-0.0052	8	1	8	7	0	7
5017.0006	5017.0005	0.0001	6	3	4	5	2	4
5139.6267	5139.6256	0.0011	8	1	7	7	2	6
5167.2735	5167.2719	0.0016	8	2	7	7	2	6
5197.9238	5197.9219	0.0019	8	1	7	7	1	6
5217.9016	5217.9020	-0.0004	6	4	3	5	3	2
5229.0543	5229.0534	0.0009	6	4	2	5	3	2
5270.6448	5270.6443	0.0005	6	4	3	5	3	3

5322.3850	5322.3813	0.0037	8	3	6	7	3	5
5381.2857	5381.2894	-0.0037	8	4	5	7	4	4
5445.7640	5445.7616	0.0024	8	4	4	7	4	3
5454.6674	5454.6653	0.0021	8	2	6	7	2	5
5489.3174	5489.3171	0.0003	7	2	5	6	1	5
5495.8468	5495.8464	0.0004	7	3	4	6	2	4
5539.0167	5539.0168	-0.0001	8	3	5	7	3	4
5563.1752	5563.1779	-0.0027	9	0	9	8	1	8
5563.5069	5563.5071	-0.0002	9	1	9	8	1	8
5564.0568	5564.0611	-0.0043	9	0	9	8	0	8
5564.3884	5564.3902	-0.0018	9	1	9	8	0	8
5610.4126	5610.4108	0.0018	6	5	2	5	4	1
5610.7376	5610.7353	0.0023	6	5	1	5	4	1
5612.7314	5612.7324	-0.0010	6	5	2	5	4	2
5613.0581	5613.0568	0.0013	6	5	1	5	4	2
5766.9576	5766.9572	0.0004	7	3	5	6	2	5
5767.4296	5767.4288	0.0008	9	1	8	8	2	7
5779.7029	5779.7019	0.0010	9	2	8	8	2	7
5795.0770	5795.0752	0.0018	9	1	8	8	1	7
5859.2021	5859.1985	0.0036	7	4	3	6	3	3
5960.1608	5960.1598	0.0010	9	3	7	8	3	6
5960.7588	5960.7601	-0.0013	7	4	4	6	3	4
5968.4750	5968.4793	-0.0043	6	6	0	5	5	0
5968.4875	5968.5061	-0.0186	6	6	1	5	5	1
6065.6939	6065.6973	-0.0034	9	2	7	8	2	6
6169.4057	6169.3975	0.0082	10	0	10	9	1	9
6169.5162	6169.5178	-0.0016	10	1	10	9	1	9
6169.7220	6169.7267	-0.0047	10	0	10	9	0	9
6169.8577	6169.8469	0.0108	10	1	10	9	0	9
6175.0702	6175.0677	0.0025	9	4	5	8	4	4
6219.5838	6219.5858	-0.0020	8	3	5	7	2	5
6270.8410	6270.8394	0.0016	7	5	3	6	4	2
6272.7298	6272.7320	-0.0022	7	5	2	6	4	2
6281.9908	6281.9908	-0.0000	7	5	3	6	4	3
6283.8820	6283.8834	-0.0014	7	5	2	6	4	3
6340.8844	6340.8845	-0.0001	8	2	6	7	1	6

6383.5530	6383.5532	-0.0002	10	1	9	9	2	8
6388.7478	6388.7488	-0.0010	10	2	9	9	2	8
6395.8263	6395.8262	0.0001	10	1	9	9	1	8
6539.9712	6539.9684	0.0028	8	3	6	7	2	6
6587.7234	6587.7193	0.0041	10	3	8	9	3	7
6636.3726	6636.3713	0.0013	7	6	1	6	5	1
6636.6539	6636.6547	-0.0008	7	6	2	6	5	2
6658.7360	6658.7381	-0.0021	10	2	8	9	2	7
6668.2852	6668.2854	-0.0002	8	4	5	7	3	5
6713.0319	6713.0358	-0.0039	10	4	7	9	4	6
6728.1799	6728.1779	0.0020	10	6	5	9	6	4
6732.0107	6732.0145	-0.0038	10	6	4	9	6	3
6743.4487	6743.4433	0.0054	10	5	6	9	5	5
6775.4281	6775.4303	-0.0022	11	1	11	10	1	10
6775.5013	6775.5073	-0.0060	11	0	11	10	0	10
6895.9436	6895.9411	0.0025	10	3	7	9	3	6
6923.8183	6923.8151	0.0032	8	5	3	7	4	3
6954.1622	6954.1653	-0.0031	8	5	4	7	4	4
6992.9141	6992.9186	-0.0045	7	7	1	6	6	1
6992.9141	6992.9158	-0.0017	7	7	0	6	6	0
6995.9594	6995.9596	-0.0002	11	2	10	10	2	9
6996.7485	6996.7481	0.0004	9	3	6	8	2	6
6999.0324	6999.0328	-0.0004	11	1	10	10	1	9
7206.8593	7206.8580	0.0013	11	3	9	10	3	8
7208.6593	7208.6600	-0.0007	9	2	7	8	1	7
7248.0115	7248.0057	0.0058	11	2	9	10	2	8
7302.2670	7302.2587	0.0083	8	6	3	7	5	2
7302.5406	7302.5396	0.0010	8	6	2	7	5	2
7304.1521	7304.1513	0.0008	8	6	3	7	5	3
7304.4380	7304.4322	0.0058	8	6	2	7	5	3
7332.8606	7332.8563	0.0043	9	3	7	8	2	7
7363.4804	7363.4906	-0.0102	11	4	8	10	4	7
7393.3226	7393.3242	-0.0016	11	7	5	10	7	4
7394.0273	7394.0260	0.0013	11	7	4	10	7	3
7397.5087	7397.5090	-0.0003	9	4	6	8	3	6
7414.6135	7414.6100	0.0035	11	6	6	10	6	5

7422.9991	7422.9996	-0.0005	11	5	7	10	5	6
7425.9905	7425.9960	-0.0055	11	6	5	10	6	4
7515.1133	7515.1117	0.0016	11	5	6	10	5	5
7527.3979	7527.3986	-0.0007	11	3	8	10	3	7
7530.7354	7530.7407	-0.0053	9	5	5	8	4	4
7601.4368	7601.4390	-0.0022	12	1	11	11	2	10
7602.2804	7602.2820	-0.0016	12	2	11	11	2	10
7603.5602	7603.5613	-0.0011	12	1	11	11	1	10
7604.4036	7604.4043	-0.0007	12	2	11	11	1	10
7633.4078	7633.4159	-0.0081	9	5	5	8	4	5
7792.1559	7792.1527	0.0032	10	4	6	9	3	6
7819.9371	7819.9359	0.0012	12	3	10	11	3	9
7826.9909	7826.9920	-0.0011	10	3	7	9	2	7
7841.2673	7841.2666	0.0007	12	2	10	11	2	9
7964.6235	7964.6231	0.0004	9	6	3	8	5	3
7971.1148	7971.1163	-0.0015	9	6	4	8	5	4
7972.4767	7972.4760	0.0007	9	6	3	8	5	4
7987.1375	7987.1493	-0.0118	13	1	13	12	1	12
7987.1625	7987.1593	0.0032	13	0	13	12	0	12

4.5 [4]RS

Supplementary Table 8: Observed and calculated rotational transitions (MHz) for the [4]RS dimer.

Observed	Calculated	Obs-Calc	J′	K_a'	K_c'	J″	K_a''	K_c''
2003.2802	2003.2776	0.0026	2	2	1	1	1	0
2004.4765	2004.4752	0.0013	2	2	0	1	1	0
2023.1618	2023.1619	-0.0001	2	2	1	1	1	1
2024.3580	2024.3595	-0.0015	2	2	0	1	1	1
2095.0716	2095.0728	-0.0012	3	1	3	2	0	2
2206.2569	2206.2545	0.0024	11	5	7	11	4	8
2211.0112	2211.0156	-0.0044	10	5	6	10	4	7
2211.1190	2211.1339	-0.0149	10	5	5	10	4	7
2214.3317	2214.3328	-0.0011	3	1	2	2	0	2

2215.0438	2215.0294	0.0144	9	5	5	9	4	6
2215.0646	2215.0690	-0.0044	9	5	4	9	4	6
2337.9789	2337.9802	-0.0013	4	0	4	3	1	3
2500.5524	2500.5564	-0.0040	4	1	4	3	1	3
2530.8671	2530.8666	0.0005	4	0	4	3	0	3
2541.6595	2541.6585	0.0010	4	2	3	3	2	2
2544.8523	2544.8577	-0.0054	4	3	2	3	3	1
2545.1330	2545.1281	0.0049	4	3	1	3	3	0
2553.3832	2553.3796	0.0036	4	2	2	3	2	1
2577.3336	2577.3299	0.0037	5	1	4	4	2	3
2579.8226	2579.8218	0.0008	4	1	3	3	1	2
2619.0407	2619.0406	0.0001	3	2	2	2	1	1
2624.9941	2624.9999	-0.0058	3	2	1	2	1	1
2678.6913	2678.6934	-0.0021	3	2	2	2	1	2
2684.6536	2684.6528	0.0008	3	2	1	2	1	2
2693.4459	2693.4428	0.0031	4	1	4	3	0	3
2794.3802	2794.3806	-0.0004	5	0	5	4	1	3
2891.9669	2891.9682	-0.0013	4	1	3	3	0	3
2992.9047	2992.9061	-0.0014	5	0	5	4	1	4
3123.7251	3123.7280	-0.0029	5	1	5	4	1	4
3143.0096	3143.0036	0.0060	3	3	1	2	2	0
3143.0500	3143.0488	0.0012	3	3	0	2	2	0
3155.4830	3155.4822	0.0008	5	0	5	4	0	4
3175.5685	3175.5649	0.0036	5	2	4	4	2	3
3180.8913	3180.9018	-0.0105	5	4	1	4	4	0
3180.8913	3180.8905	0.0008	5	4	2	4	4	1
3181.8843	3181.8892	-0.0049	5	3	3	4	3	2
3182.8265	3182.8310	-0.0045	5	3	2	4	3	1
3198.2944	3198.2946	-0.0002	5	2	3	4	2	2
3206.1750	3206.1753	-0.0003	11	7	5	11	6	5
3206.1750	3206.1754	-0.0004	11	7	4	11	6	5
3208.2346	3208.2376	-0.0030	10	7	4	10	6	5
3208.2346	3208.2357	-0.0011	10	7	4	10	6	4
3208.2346	3208.2377	-0.0031	10	7	3	10	6	5
3208.2346	3208.2357	-0.0011	10	7	3	10	6	4
3209.7792	3209.7781	0.0011	9	7	3	9	6	4

3209.7792	3209.7781	0.0011	9	7	2	9	6	4
3209.7792	3209.7777	0.0015	9	7	3	9	6	3
3209.7792	3209.7777	0.0015	9	7	2	9	6	3
3210.8988	3210.8981	0.0007	8	7	2	8	6	3
3210.8988	3210.8980	0.0008	8	7	2	8	6	2
3210.8988	3210.8981	0.0007	8	7	1	8	6	3
3210.8988	3210.8980	0.0008	8	7	1	8	6	2
3211.6845	3211.6822	0.0023	7	7	0	7	6	1
3211.6845	3211.6822	0.0023	7	7	1	7	6	2
3211.6845	3211.6822	0.0023	7	7	1	7	6	1
3211.6845	3211.6822	0.0023	7	7	0	7	6	2
3222.2013	3222.2050	-0.0037	5	1	4	4	1	3
3224.6948	3224.6969	-0.0021	4	2	3	3	1	2
3242.3816	3242.3773	0.0043	4	2	2	3	1	2
3264.3904	3264.3867	0.0037	6	1	5	5	2	4
3286.3054	3286.3041	0.0013	5	1	5	4	0	4
3343.9642	3343.9569	0.0073	4	2	3	3	1	3
3348.2038	3348.1854	0.0184	6	0	6	5	1	4
3361.6366	3361.6373	-0.0007	4	2	2	3	1	3
3583.3049	3583.3065	-0.0016	5	1	4	4	0	4
3645.1834	3645.1878	-0.0044	6	0	6	5	1	5
3692.8754	3692.8724	0.0030	15	8	7	15	7	8
3692.8754	3692.8809	-0.0055	15	8	7	15	7	9
3692.8754	3692.8723	0.0031	15	8	8	15	7	8
3692.8754	3692.8807	-0.0053	15	8	8	15	7	9
3696.2398	3696.2426	-0.0028	14	8	7	14	7	8
3696.2398	3696.2396	0.0002	14	8	7	14	7	7
3696.2398	3696.2427	-0.0029	14	8	6	14	7	8
3696.2398	3696.2396	0.0002	14	8	6	14	7	7
3698.9658	3698.9655	0.0003	13	8	5	13	7	6
3698.9658	3698.9654	0.0004	13	8	6	13	7	6
3698.9658	3698.9665	-0.0007	13	8	6	13	7	7
3698.9658	3698.9665	-0.0007	13	8	5	13	7	7
3701.1430	3701.1403	0.0027	12	8	5	12	7	6
3702.8528	3702.8450	0.0078	11	8	4	11	7	5
3704.1549	3704.1541	0.0008	10	8	3	10	7	4

3705.1319	3705.1346	-0.0027	9	8	2	9	7	3
3705.8349	3705.8469	-0.0120	8	8	1	8	7	2
3745.8135	3745.8095	0.0040	6	1	6	5	1	5
3776.0125	3776.0097	0.0028	6	0	6	5	0	5
3776.1536	3776.1504	0.0032	4	3	2	3	2	1
3776.4600	3776.4660	-0.0060	4	3	1	3	2	1
3782.1030	3782.1097	-0.0067	4	3	2	3	2	2
3782.4201	3782.4253	-0.0052	4	3	1	3	2	2
3808.4817	3808.4760	0.0057	6	2	5	5	2	4
3816.8495	3816.8603	-0.0108	6	5	1	5	5	0
3816.8495	3816.8599	-0.0104	6	5	2	5	5	1
3817.9250	3817.9125	0.0125	6	4	3	5	4	2
3817.9559	3817.9632	-0.0073	6	4	2	5	4	1
3819.2469	3819.2440	0.0029	6	3	4	5	3	3
3820.4418	3820.4400	0.0018	5	2	4	4	1	3
3821.7313	3821.7323	-0.0010	6	3	3	5	3	2
3846.2636	3846.2638	-0.0002	6	2	4	5	2	3
3860.8515	3860.8500	0.0015	5	2	3	4	1	3
3862.6257	3862.6217	0.0040	6	1	5	5	1	4
3876.6333	3876.6314	0.0019	6	1	6	5	0	5
3878.1596	3878.1576	0.0020	7	1	6	6	2	4
3878.7156	3878.7157	-0.0001	7	0	7	6	1	5
3956.3624	3956.3554	0.0070	7	1	6	6	2	5
4018.9651	4018.9655	-0.0004	5	2	4	4	1	4
4059.3699	4059.3755	-0.0056	5	2	3	4	1	4
4193.9868	4193.9814	0.0054	14	9	5	14	8	6
4193.9868	4193.9815	0.0053	14	9	5	14	8	7
4193.9868	4193.9815	0.0053	14	9	6	14	8	7
4193.9868	4193.9814	0.0054	14	9	6	14	8	6
4195.8215	4195.8191	0.0024	13	9	4	13	8	5
4195.8215	4195.8192	0.0023	13	9	4	13	8	6
4195.8215	4195.8191	0.0024	13	9	5	13	8	5
4195.8215	4195.8192	0.0023	13	9	5	13	8	6
4197.2881	4197.2861	0.0020	12	9	4	12	8	4
4197.2881	4197.2861	0.0020	12	9	3	12	8	4
4197.2881	4197.2862	0.0019	12	9	4	12	8	5

4197.2881	4197.2862	0.0019	12	9	3	12	8	5
4198.4461	4198.4364	0.0097	11	9	3	11	8	4
4198.4461	4198.4364	0.0097	11	9	3	11	8	3
4198.4461	4198.4364	0.0097	11	9	2	11	8	4
4198.4461	4198.4364	0.0097	11	9	2	11	8	3
4199.3212	4199.3194	0.0018	10	9	2	10	8	3
4199.3212	4199.3194	0.0018	10	9	2	10	8	2
4199.3212	4199.3194	0.0018	10	9	1	10	8	2
4199.3212	4199.3194	0.0018	10	9	1	10	8	3
4199.9883	4199.9800	0.0083	9	9	1	9	8	2
4199.9883	4199.9800	0.0083	9	9	1	9	8	1
4199.9883	4199.9800	0.0083	9	9	0	9	8	2
4199.9883	4199.9800	0.0083	9	9	0	9	8	1
4273.8750	4273.8778	-0.0028	4	4	0	3	3	0
4273.9250	4273.9230	0.0020	4	4	0	3	3	1
4290.4471	4290.4461	0.0010	6	1	5	5	0	5
4292.5493	4292.5304	0.0189	7	0	7	6	1	6
4366.7737	4366.7775	-0.0038	7	1	7	6	1	6
4386.3491	4386.3475	0.0016	8	0	8	7	1	6
4393.1511	4393.1521	-0.0010	7	0	7	6	0	6
4404.6567	4404.6600	-0.0033	5	3	3	4	2	2
4405.9173	4405.9174	-0.0001	5	3	2	4	2	2
4406.6999	4406.7110	-0.0111	6	2	5	5	1	4
4422.3383	4422.3404	-0.0021	5	3	3	4	2	3
4423.5945	4423.5978	-0.0033	5	3	2	4	2	3
4440.2107	4440.2107	-0.0000	7	2	6	6	2	5
4452.8417	4452.8430	-0.0013	7	6	2	6	6	1
4452.8417	4452.8430	-0.0013	7	6	1	6	6	0
4453.7398	4453.7369	0.0029	7	5	2	6	5	1
4455.3675	4455.3702	-0.0027	7	4	4	6	4	3
4455.5380	4455.5383	-0.0003	7	4	3	6	4	2
4456.7974	4456.7920	0.0054	7	3	5	6	3	4
4462.3082	4462.3049	0.0033	7	3	4	6	3	3
4467.3964	4467.3991	-0.0027	7	1	7	6	0	6
4484.9066	4484.9089	-0.0023	6	2	4	5	1	4
4496.3516	4496.3532	-0.0016	7	2	5	6	2	4

4500.4418	4500.4447	-0.0029	7	1	6	6	1	5
4516.7704	4516.7772	-0.0068	8	1	7	7	2	5
4642.1908	4642.1885	0.0023	10	3	8	9	4	5
4651.1161	4651.1176	-0.0015	8	1	7	7	2	6
4693.4543	4693.4600	-0.0057	11	10	2	11	9	3
4693.4543	4693.4600	-0.0057	11	10	2	11	9	2
4693.4543	4693.4600	-0.0057	11	10	1	11	9	3
4693.4543	4693.4600	-0.0057	11	10	1	11	9	2
4694.0821	4694.0814	0.0007	10	10	1	10	9	2
4694.0821	4694.0814	0.0007	10	10	1	10	9	1
4694.0821	4694.0814	0.0007	10	10	0	10	9	2
4694.0821	4694.0814	0.0007	10	10	0	10	9	1
4703.7165	4703.7135	0.0030	6	2	5	5	1	5
4710.1736	4710.1626	0.0110	9	2	7	8	3	6
4713.8958	4713.9074	-0.0116	10	3	7	9	4	6
4781.9088	4781.9113	-0.0025	6	2	4	5	1	5
4909.6312	4909.6515	-0.0203	5	4	1	4	3	1
4909.9447	4909.9544	-0.0097	5	4	2	4	3	2
4933.8291	4933.8294	-0.0003	8	0	8	7	1	7
4984.3002	4984.3000	0.0002	7	2	6	6	1	5
4986.6796	4986.6787	0.0009	8	1	8	7	1	7
5008.0804	5008.0765	0.0039	8	0	8	7	0	7
5014.8824	5014.8810	0.0014	7	1	6	6	0	6
5025.6102	5025.6095	0.0007	6	3	4	5	2	3
5029.3537	5029.3551	-0.0014	6	3	3	5	2	3
5060.9229	5060.9258	-0.0029	8	1	8	7	0	7
5066.0189	5066.0195	-0.0006	6	3	4	5	2	4
5069.7656	5069.7652	0.0004	6	3	3	5	2	4
5070.6088	5070.6074	0.0014	8	2	7	7	2	6
5088.8335	5088.8300	0.0035	8	7	1	7	7	0
5088.8335	5088.8300	0.0035	8	7	2	7	7	1
5089.6111	5089.6142	-0.0031	8	6	2	7	6	1
5090.9529	5090.9555	-0.0026	8	5	3	7	5	2
5093.3051	5093.3014	0.0037	8	4	5	7	4	4
5093.7632	5093.7604	0.0028	8	4	4	7	4	3
5094.3361	5094.3333	0.0028	8	3	6	7	3	5

5105.0944	5105.0932	0.0012	8	3	5	7	3	4
5118.6427	5118.6403	0.0024	7	2	5	6	1	5
5134.9713	5134.9729	-0.0016	8	1	7	7	1	6
5135.1556	5135.1562	-0.0006	9	1	8	8	2	6
5147.1394	5147.1365	0.0029	8	2	6	7	2	5
5186.7992	5186.8050	-0.0058	13	11	2	13	10	3
5186.7992	5186.8050	-0.0058	13	11	2	13	10	4
5186.7992	5186.8050	-0.0058	13	11	3	13	10	4
5186.7992	5186.8050	-0.0058	13	11	3	13	10	3
5187.5555	5187.5594	-0.0039	12	11	1	12	10	3
5187.5555	5187.5594	-0.0039	12	11	2	12	10	2
5187.5555	5187.5594	-0.0039	12	11	2	12	10	3
5187.5555	5187.5594	-0.0039	12	11	1	12	10	2
5188.1488	5188.1495	-0.0007	11	11	1	11	10	2
5188.1488	5188.1495	-0.0007	11	11	0	11	10	2
5188.1488	5188.1495	-0.0007	11	11	1	11	10	1
5188.1488	5188.1495	-0.0007	11	11	0	11	10	1
5346.0259	5346.0256	0.0003	9	1	8	8	2	7
5398.1120	5398.1147	-0.0027	7	2	6	6	1	6
5404.1385	5404.1350	0.0035	5	5	1	4	4	1
5532.4599	5532.4550	0.0049	7	2	5	6	1	6
5544.7170	5544.7203	-0.0033	6	4	3	5	3	2
5544.7878	5544.7837	0.0041	6	4	2	5	3	2
5545.9706	5545.9777	-0.0071	6	4	3	5	3	3
5546.0431	5546.0410	0.0021	6	4	2	5	3	3
5554.4641	5554.4627	0.0014	8	2	7	7	1	6
5569.1056	5569.1019	0.0037	9	0	9	8	1	8
5605.6162	5605.6151	0.0011	9	1	9	8	1	8
5621.9501	5621.9512	-0.0011	9	0	9	8	0	8
5636.1370	5636.1376	-0.0006	7	3	5	6	2	4
5645.3951	5645.3962	-0.0011	7	3	4	6	2	4
5658.4614	5658.4644	-0.0030	9	1	9	8	0	8
5699.5283	5699.5332	-0.0049	9	2	8	8	2	7
5714.3410	5714.3354	0.0056	7	3	5	6	2	5
5723.5966	5723.5941	0.0025	7	3	4	6	2	5
5724.8208	5724.8141	0.0067	9	8	2	8	8	1

5724.8208	5724.8141	0.0067	9	8	1	8	8	0
5725.5323	5725.5264	0.0059	9	7	2	8	7	1
5726.6559	5726.6467	0.0092	9	6	3	8	6	2
5728.5600	5728.5710	-0.0110	9	5	4	8	5	3
5729.5726	5729.5696	0.0030	10	1	9	9	2	7
5731.6208	5731.6144	0.0064	9	3	7	8	3	6
5731.7153	5731.7150	0.0003	9	4	6	8	4	5
5732.8094	5732.8052	0.0042	9	4	5	8	4	4
5750.6316	5750.6277	0.0039	9	3	6	8	3	5
5756.7100	5756.7019	0.0081	8	1	7	7	0	7
5765.3320	5765.3321	-0.0001	8	2	6	7	1	6
5765.5146	5765.5154	-0.0008	9	1	8	8	1	7
5797.1465	5797.1438	0.0027	9	2	7	8	2	6
6038.0536	6038.0497	0.0039	10	1	9	9	2	8
6040.0939	6040.0917	0.0022	6	5	2	5	4	1
6101.9521	6101.9446	0.0075	8	2	7	7	1	7
6119.0258	6119.0230	0.0028	9	2	8	8	1	7
6178.3595	6178.3582	0.0013	7	4	4	6	3	3
6178.5928	6178.5896	0.0032	7	4	3	6	3	3
6182.1082	6182.1039	0.0043	7	4	4	6	3	4
6182.3409	6182.3353	0.0056	7	4	3	6	3	4
6199.0972	6199.0960	0.0012	10	0	10	9	1	9
6223.7306	6223.7225	0.0081	10	1	10	9	1	9
6234.1042	6234.1177	-0.0135	8	3	6	7	2	5
6235.6007	6235.6092	-0.0085	10	0	10	9	0	9
6254.1267	6254.1362	-0.0095	8	3	5	7	2	5
6260.2345	6260.2357	-0.0012	10	1	10	9	0	9
6297.3818	6297.3727	0.0091	11	1	10	10	2	8
6312.8125	6312.8140	-0.0015	8	2	6	7	1	7
6326.8851	6326.8947	-0.0096	10	2	9	9	2	8
6360.7857	6360.7913	-0.0056	10	9	2	9	9	1
6360.7857	6360.7913	-0.0056	10	9	1	9	9	0
6361.4516	6361.4519	-0.0003	10	8	2	9	8	1
6362.4340	6362.4325	0.0015	10	7	3	9	7	2
6363.9745	6363.9744	0.0001	10	6	4	9	6	3
6366.5668	6366.5664	0.0004	10	5	6	9	5	5

6366.6470	6366.6450	0.0020	10	5	5	9	5	4
6368.3506	6368.3498	0.0008	10	3	8	9	3	7
6368.4568	6368.4581	-0.0013	8	3	6	7	2	6
6370.5798	6370.5802	-0.0004	10	4	7	9	4	6
6372.9086	6372.9079	0.0007	10	4	6	9	4	5
6388.4756	6388.4765	-0.0009	8	3	5	7	2	6
6391.5603	6391.5573	0.0030	10	1	9	9	1	8
6399.2634	6399.2563	0.0071	10	3	7	9	3	6
6427.5079	6427.5031	0.0048	9	2	7	8	1	7
6445.1793	6445.1806	-0.0013	10	2	8	9	2	7
6514.1357	6514.1408	-0.0051	9	1	8	8	0	8
6534.3460	6534.3432	0.0028	6	6	1	5	5	1
6675.8750	6675.8658	0.0092	7	5	2	6	4	2
6675.9158	6675.9266	-0.0108	7	5	3	6	4	3
6680.4011	6680.4023	-0.0012	10	2	9	9	1	8
6724.1403	6724.1386	0.0017	11	1	10	10	2	9
6809.3526	6809.3547	-0.0021	8	4	5	7	3	4
6810.0454	6810.0451	0.0003	8	4	4	7	3	4
6814.7923	6814.7991	-0.0068	9	2	8	8	1	8
6818.6067	6818.6133	-0.0066	8	4	5	7	3	5
6819.3041	6819.3038	0.0003	8	4	4	7	3	5
6824.8520	6824.8584	-0.0064	11	0	11	10	1	10
6841.1594	6841.1503	0.0091	11	1	11	10	1	10
6849.4815	6849.4849	-0.0034	11	0	11	10	0	10
6857.6215	6857.6274	-0.0059	9	3	6	8	2	6
6865.7783	6865.7768	0.0015	11	1	11	10	0	10
6952.6528	6952.6469	0.0059	11	2	10	10	2	9
6997.3732	6997.3800	-0.0068	11	9	2	10	9	1
6998.2677	6998.2629	0.0048	11	8	3	10	8	2
6999.5688	6999.5721	-0.0033	11	7	4	10	7	3
7001.6288	7001.6279	0.0009	11	6	6	10	6	5
7001.6306	7001.6325	-0.0019	11	6	5	10	6	4
7004.2427	7004.2465	-0.0038	11	3	9	10	3	8
7005.0561	7005.0558	0.0003	11	5	7	10	5	6
7005.2489	7005.2509	-0.0020	11	5	6	10	5	5
7009.8165	7009.8169	-0.0004	11	4	8	10	4	7

7012.9837	7012.9837	0.0000	11	1	10	10	1	9
7014.3764	7014.3788	-0.0024	11	4	7	10	4	6
7029.4645	7029.4651	-0.0006	9	3	7	8	2	7
7050.9172	7050.9146	0.0026	11	3	8	10	3	7
7068.4868	7068.4969	-0.0101	9	3	6	8	2	7
7090.3710	7090.3737	-0.0027	11	2	9	10	2	8
7107.1644	7107.1683	-0.0039	10	2	8	9	1	8
7170.3222	7170.3262	-0.0040	7	6	2	6	5	2
7241.4838	7241.4919	-0.0081	11	2	10	10	1	9
7311.3083	7311.2830	0.0253	8	5	3	7	4	3
7311.5422	7311.5145	0.0277	8	5	3	7	4	4
7389.8093	7389.8016	0.0077	10	3	8	9	2	7
7401.7506	7401.7469	0.0037	12	1	11	11	2	10
7421.6467	7421.6482	-0.0015	13	2	11	12	3	9
7435.9798	7435.9766	0.0032	9	4	6	8	3	5
7437.7615	7437.7572	0.0043	9	4	5	8	3	5
7447.4290	7447.4313	-0.0023	12	0	12	11	1	11
7455.9909	7455.9950	-0.0041	9	4	6	8	3	6
7457.7756	7457.7757	-0.0001	9	4	5	8	3	6
7458.0441	7458.0428	0.0013	12	1	12	11	1	11
7459.7452	7459.7398	0.0054	10	3	7	9	2	7
7463.7214	7463.7232	-0.0018	12	0	12	11	0	11
7474.3319	7474.3347	-0.0028	12	1	12	11	0	11
7536.0750	7536.0787	-0.0037	10	2	9	9	1	9
7576.8013	7576.7995	0.0018	12	2	11	11	2	10
7630.2680	7630.2552	0.0128	12	1	11	11	1	10
7636.9821	7636.9696	0.0125	12	7	5	11	7	4
7639.0188	7639.0259	-0.0071	12	3	10	11	3	9
7639.6469	7639.6451	0.0018	12	6	7	11	6	6
7639.6568	7639.6580	-0.0012	12	6	6	11	6	5
7644.0456	7644.0391	0.0065	12	5	8	11	5	7
7644.4793	7644.4802	-0.0009	12	5	7	11	5	6
7649.2825	7649.2901	-0.0076	12	4	9	11	4	8
7657.6222	7657.6117	0.0105	12	4	8	11	4	7
7664.5287	7664.5252	0.0035	7	7	0	6	6	0
7698.2768	7698.2816	-0.0048	10	3	8	9	2	8

7704.9346	7704.9438	-0.0092	12	3	9	11	3	8
7732.0538	7732.0546	-0.0008	12	2	10	11	2	9
7768.2222	7768.2199	0.0023	10	3	7	9	2	8
7805.3097	7805.3078	0.0019	12	2	11	11	1	10
7805.9903	7805.9847	0.0056	11	2	9	10	1	9
7806.2030	7806.2031	-0.0001	8	6	2	7	5	2
7946.0730	7946.0936	-0.0206	9	5	4	8	4	4
7946.7707	7946.7840	-0.0133	9	5	4	8	4	5
7948.8672	7948.8674	-0.0002	11	3	9	10	2	8

4.6 [3]RR singly substituted ^{13}C isotopologs

Supplementary Table 9: Observed and calculated rotational transitions (MHz) for the [3]RR dimer. (^{12}C1 $\rightarrow$^{13}C) Fitted rotational parameters: A=517.81675(13) MHz, B=349.06709(777) MHz, C=322.38336(815) MHz.

Observed	Calculated	Obs-Calc	J′	K_a'	K_c'	J″	K_a''	K_c''
7010.7361	7010.7361	-0.0000	8	5	3	7	4	3
7012.4191	7012.4192	-0.0001	8	5	4	7	4	4
6704.3678	6704.3699	-0.0021	7	6	2	6	5	2
6704.3678	6704.3638	0.0040	7	6	1	6	5	1
6032.1053	6032.1090	-0.0037	6	6	1	5	5	1
6032.1053	6032.1084	-0.0031	6	6	0	5	5	0
7739.9688	7739.9696	-0.0008	8	7	2	7	6	2
7739.9688	7739.9692	-0.0004	8	7	1	7	6	1
7067.7063	7067.7036	0.0027	7	7	1	6	6	1
7067.7063	7067.7036	0.0027	7	7	0	6	6	0

Supplementary Table 10: Observed and calculated rotational transitions (MHz) for the [3]RR dimer. (^{12}C2 $\rightarrow$^{13}C) Fitted rotational parameters: A=516.90261(25) MHz, B=349.3484(152) MHz, C=322.2547(150) MHz.

Observed	Calculated	Obs-Calc	J′	K_a′	K_c′	J″	K_a″	K_c″
7728.3617	7728.3658	-0.0041	8	7	2	7	6	2
7728.3617	7728.3654	-0.0037	8	7	1	7	6	1
7055.9147	7055.9137	0.0010	7	7	1	6	6	1
7055.9147	7055.9136	0.0011	7	7	0	6	6	0
7003.0263	7003.0264	-0.0001	8	5	3	7	4	3
6694.5937	6694.5933	0.0004	7	6	2	6	5	2
6694.5937	6694.5866	0.0071	7	6	1	6	5	1
6022.1466	6022.1473	-0.0007	6	6	1	5	5	1
6022.1466	6022.1466	-0.0000	6	6	0	5	5	0

Supplementary Table 11: Observed and calculated rotational transitions (MHz) for the [3]RR dimer. (^{12}C7 $\rightarrow$^{13}C) Fitted rotational parameters: A=519.614233(53) MHz, B=349.38403(297) MHz, C=321.94284(312) MHz.

Observed	Calculated	Obs-Calc	J′	K_a′	K_c′	J″	K_a″	K_c″
7026.4748	7026.4748	-0.0000	8	5	3	7	4	3
7028.2962	7028.2958	0.0004	8	5	4	7	4	4
7763.2168	7763.2169	-0.0001	8	7	2	7	6	2
7763.2168	7763.2165	0.0003	8	7	1	7	6	1
7091.0308	7091.0316	-0.0008	7	7	1	6	6	1
7091.0308	7091.0316	-0.0008	7	7	0	6	6	0
6724.0125	6724.0141	-0.0016	7	6	1	6	5	1
6051.8430	6051.8419	0.0011	6	6	1	5	5	1
6051.8430	6051.8413	0.0017	6	6	0	5	5	0

Supplementary Table 12: Observed and calculated rotational transitions (MHz) for the [3]RR dimer. (^{12}C8 $\rightarrow^{13}$C) Fitted rotational parameters: A=518.95060(12) MHz, B=349.24068(635) MHz, C=321.43375(667) MHz.

Observed	Calculated	Obs-Calc	J′	K_a′	K_c′	J″	K_a″	K_c″
7753.6528	7753.6530	-0.0002	8	7	2	7	6	2
7753.6528	7753.6525	0.0003	8	7	1	7	6	1
7018.2056	7018.2056	-0.0000	8	5	3	7	4	3
7020.1336	7020.1318	0.0018	8	5	4	7	4	4
7082.0931	7082.0926	0.0005	7	7	1	6	6	1
7082.0931	7082.0926	0.0005	7	7	0	6	6	0
6715.7704	6715.7760	-0.0056	7	6	1	6	5	1
6044.2312	6044.2301	0.0011	6	6	1	5	5	1
6044.2312	6044.2294	0.0018	6	6	0	5	5	0

Supplementary Table 13: Observed and calculated rotational transitions (MHz) for the [3]RR dimer. (^{12}C9 $\rightarrow^{13}$C) Fitted rotational parameters: A=519.19111(14) MHz, B=349.05874(793) MHz, C=321.37368(832) MHz.

Observed	Calculated	Obs-Calc	J′	K_a′	K_c′	J″	K_a″	K_c″
7019.5294	7019.5295	-0.0001	8	5	3	7	4	3
7021.4157	7021.4128	0.0029	8	5	4	7	4	4
7085.0932	7085.0927	0.0005	7	7	1	6	6	1
7085.0932	7085.0926	0.0006	7	7	0	6	6	0
6046.7518	6046.7492	0.0026	6	6	1	5	5	1
6046.7518	6046.7485	0.0033	6	6	0	5	5	0
7756.3984	7756.4005	-0.0021	8	7	2	7	6	2
7756.3984	7756.4000	-0.0016	8	7	1	7	6	1
6718.0379	6718.0430	-0.0051	7	6	1	6	5	1

Supplementary Table 14: Observed and calculated rotational transitions (MHz) for the [3]RR dimer. (^{12}C10 →^{13}C) Fitted rotational parameters: A=518.12840(18) MHz, B=348.29519(973) MHz, C=320.6653(102) MHz.

Observed	Calculated	Obs-Calc	J′	K_a′	K_c′	J″	K_a″	K_c″
7006.6919	7006.6910	0.0009	8	5	4	7	4	4
7004.8126	7004.8126	-0.0000	8	5	3	7	4	3
7070.5369	7070.5403	-0.0034	7	7	1	6	6	1
7070.5369	7070.5403	-0.0034	7	7	0	6	6	0
6704.1463	6704.1492	-0.0029	7	6	2	6	5	2
6034.3273	6034.3223	0.0050	6	6	1	5	5	1
6034.3273	6034.3216	0.0057	6	6	0	5	5	0
7740.3737	7740.3741	-0.0004	8	7	2	7	6	2
7740.3737	7740.3737	0.0000	8	7	1	7	6	1

Supplementary Table 15: Observed and calculated rotational transitions (MHz) for the [3]RR dimer. (^{12}C12 →^{13}C) Fitted rotational parameters: A=518.49049(18) MHz, B=347.8564(106) MHz, C=320.7918(111) MHz.

Observed	Calculated	Obs-Calc	J′	K_a′	K_c′	J″	K_a″	K_c″
7008.7154	7008.7176	-0.0022	8	5	4	7	4	4
7006.9967	7006.9966	0.0001	8	5	3	7	4	3
7075.0712	7075.0693	0.0019	7	7	1	6	6	1
7075.0712	7075.0693	0.0019	7	7	0	6	6	0
6707.6004	6707.5948	0.0056	7	6	1	6	5	1
6038.1219	6038.1272	-0.0053	6	6	1	5	5	1
6038.1219	6038.1266	-0.0047	6	6	0	5	5	0
7744.5492	7744.5487	0.0005	8	7	2	7	6	2
7744.5492	7744.5484	0.0008	8	7	1	7	6	1

Supplementary Table 16: Observed and calculated rotational transitions (MHz) for the [3]RR dimer. (^{12}C14 $\rightarrow ^{13}$C) Fitted rotational parameters: A=518.07470(48) MHz, B=347.1942(290) MHz, C=320.6402(304) MHz.

Observed	Calculated	Obs-Calc	J′	K_a′	K_c′	J″	K_a″	K_c″
6033.1213	6033.1284	-0.0071	6	6	1	5	5	1
6033.1213	6033.1279	-0.0066	6	6	0	5	5	0
6701.7682	6701.7547	0.0135	7	6	2	6	5	2
6701.7682	6701.7490	0.0192	7	6	1	6	5	1
7069.2363	7069.2388	-0.0025	7	7	1	6	6	1
7069.2363	7069.2388	-0.0025	7	7	0	6	6	0
7002.0057	7002.0156	-0.0099	8	5	4	7	4	4
7000.4167	7000.4161	0.0006	8	5	3	7	4	3
7737.8671	7737.8696	-0.0025	8	7	2	7	6	2
7737.8671	7737.8693	-0.0022	8	7	1	7	6	1

Supplementary Table 17: Observed and calculated rotational transitions (MHz) for the [3]RR dimer. (^{12}C18 $\rightarrow ^{13}$C) Fitted rotational parameters: A=518.01682(29) MHz, B=349.8807(147) MHz, C=321.5956(155) MHz.

Observed	Calculated	Obs-Calc	J′	K_a′	K_c′	J″	K_a″	K_c″
7014.6800	7014.6802	-0.0002	8	5	4	7	4	4
7012.5807	7012.5806	0.0001	8	5	3	7	4	3
7070.3696	7070.3764	-0.0068	7	7	1	6	6	1
7070.3696	7070.3764	-0.0068	7	7	0	6	6	0
7742.7835	7742.7802	0.0033	8	7	2	7	6	2
7742.7835	7742.7797	0.0038	8	7	1	7	6	1
6706.7690	6706.7767	-0.0077	7	6	2	6	5	2
6706.7690	6706.7686	0.0004	7	6	1	6	5	1
6034.3884	6034.3813	0.0071	6	6	1	5	5	1
6034.3884	6034.3805	0.0079	6	6	0	5	5	0

Supplementary Table 18: Observed and calculated rotational transitions (MHz) for the [3]RR dimer. ($^{12}C19 \rightarrow ^{13}C$) Fitted rotational parameters: A=515.55452(22) MHz, B=349.0461(101) MHz, C=319.9658(107) MHz.

Observed	Calculated	Obs-Calc	J′	K_a′	K_c′	J″	K_a″	K_c″
7707.1797	7707.1744	0.0053	8	7	2	7	6	2
7707.1797	7707.1738	0.0059	8	7	1	7	6	1
7037.1686	7037.1690	-0.0004	7	7	1	6	6	1
7037.1686	7037.1690	-0.0004	7	7	0	6	6	0
6981.7481	6981.7481	-0.0000	8	5	3	7	4	3
6984.1250	6984.1283	-0.0033	8	5	4	7	4	4
6676.0887	6676.0935	-0.0048	7	6	2	6	5	2
6676.0887	6676.0840	0.0047	7	6	1	6	5	1
6006.0931	6006.0983	-0.0052	6	6	1	5	5	1
6006.0931	6006.0974	-0.0043	6	6	0	5	5	0

Supplementary Table 19: Observed and calculated rotational transitions (MHz) for the [3]RR dimer. ($^{12}C24 \rightarrow ^{13}C$) Fitted rotational parameters: A=519.59397(18) MHz, B=349.5269(100) MHz, C=322.0227(105) MHz.

Observed	Calculated	Obs-Calc	J′	K_a′	K_c′	J″	K_a″	K_c″
7028.9125	7028.9100	0.0025	8	5	4	7	4	4
7027.0691	7027.0692	-0.0001	8	5	3	7	4	3
7090.8829	7090.8821	0.0008	7	7	1	6	6	1
7090.8829	7090.8821	0.0008	7	7	0	6	6	0
6051.7338	6051.7330	0.0008	6	6	1	5	5	1
6051.7338	6051.7323	0.0015	6	6	0	5	5	0
7763.2973	7763.2952	0.0021	8	7	2	7	6	2
7763.2973	7763.2948	0.0025	8	7	1	7	6	1
6724.1303	6724.1395	-0.0092	7	6	2	6	5	2
6724.1303	6724.1326	-0.0023	7	6	1	6	5	1

Supplementary Table 20: Observed and calculated rotational transitions (MHz) for the [3]RR dimer. (^{12}C25 $\rightarrow^{13}$C) Fitted rotational parameters: A=518.54226(28) MHz, B=349.4604(162) MHz, C=322.4305(170) MHz.

Observed	Calculated	Obs-Calc	J′	K_a′	K_c′	J″	K_a″	K_c″
7750.0965	7750.0943	0.0022	8	7	2	7	6	2
7750.0965	7750.0939	0.0026	8	7	1	7	6	1
7020.5613	7020.5571	0.0042	8	5	4	7	4	4
7018.8022	7018.8024	-0.0002	8	5	3	7	4	3
7077.3726	7077.3666	0.0060	7	7	1	6	6	1
7077.3726	7077.3666	0.0060	7	7	0	6	6	0
6713.0318	6713.0429	-0.0111	7	6	2	6	5	2
6713.0318	6713.0365	-0.0047	7	6	1	6	5	1
6040.3170	6040.3210	-0.0040	6	6	1	5	5	1
6040.3170	6040.3204	-0.0034	6	6	0	5	5	0

Supplementary Table 21: Observed and calculated rotational transitions (MHz) for the [3]RR dimer. (^{12}C26 $\rightarrow^{13}$C) Fitted rotational parameters: A=519.820096(89) MHz, B=348.50755(508) MHz, C=321.15828(533) MHz.

Observed	Calculated	Obs-Calc	J′	K_a′	K_c′	J″	K_a″	K_c″
7092.8704	7092.8711	-0.0007	7	7	1	6	6	1
7092.8704	7092.8711	-0.0007	7	7	0	6	6	0
7022.5233	7022.5233	0.0000	8	5	3	7	4	3
7024.2916	7024.2924	-0.0008	8	5	4	7	4	4
7763.3832	7763.3836	-0.0004	8	7	2	7	6	2
7763.3832	7763.3832	-0.0000	8	7	1	7	6	1
6053.2695	6053.2697	-0.0002	6	6	1	5	5	1
6053.2695	6053.2691	0.0004	6	6	0	5	5	0
6723.7743	6723.7762	-0.0019	7	6	2	6	5	2
6723.7743	6723.7697	0.0046	7	6	1	6	5	1

Supplementary Table 22: Observed and calculated rotational transitions (MHz) for the [3]RR dimer. (^{12}C27 $\rightarrow ^{13}$C) Fitted rotational parameters: A=516.71233(36) MHz, B=349.1196(196) MHz, C=321.6771(206) MHz.

Observed	Calculated	Obs-Calc	J′	K_a'	K_c'	J″	K_a''	K_c''
7000.3885	7000.3805	0.0080	8	5	4	7	4	4
6998.4825	6998.4828	-0.0003	8	5	3	7	4	3
7053.0532	7053.0492	0.0040	7	7	1	6	6	1
7053.0532	7053.0492	0.0040	7	7	0	6	6	0
7724.7088	7724.7183	-0.0095	8	7	2	7	6	2
7724.7088	7724.7179	-0.0091	8	7	1	7	6	1
6019.6690	6019.6633	0.0057	6	6	1	5	5	1
6019.6690	6019.6626	0.0064	6	6	0	5	5	0
6691.3125	6691.3185	-0.0060	7	6	1	6	5	1

Supplementary Table 23: Observed and calculated rotational transitions (MHz) for the [3]RR dimer. (^{12}C29 $\rightarrow ^{13}$C) Fitted rotational parameters: A=518.62868(17) MHz, B=348.05727(941) MHz, C=320.28212(988) MHz.

Observed	Calculated	Obs-Calc	J′	K_a'	K_c'	J″	K_a''	K_c''
7007.1430	7007.1429	0.0001	8	5	3	7	4	3
7009.0323	7009.0357	-0.0034	8	5	4	7	4	4
6708.7310	6708.7294	0.0016	7	6	2	6	5	2
6708.7310	6708.7223	0.0087	7	6	1	6	5	1
7076.7351	7076.7365	-0.0014	7	7	1	6	6	1
7076.7351	7076.7365	-0.0014	7	7	0	6	6	0
7745.9548	7745.9551	-0.0003	8	7	2	7	6	2
7745.9548	7745.9547	0.0001	8	7	1	7	6	1
6039.5156	6039.5179	-0.0023	6	6	1	5	5	1
6039.5156	6039.5172	-0.0016	6	6	0	5	5	0

Supplementary Table 24: Observed and calculated rotational transitions (MHz) for the [3]RR dimer. (^{12}C31 $\rightarrow ^{13}$C) Fitted rotational parameters: A=516.58001(28) MHz, B=348.6871(141) MHz, C=320.4353(149) MHz.

Observed	Calculated	Obs-Calc	J′	K_a′	K_c′	J″	K_a″	K_c″
6017.3934	6017.3989	-0.0055	6	6	1	5	5	1
6017.3934	6017.3982	-0.0048	6	6	0	5	5	0
6687.4342	6687.4396	-0.0054	7	6	2	6	5	2
6687.4342	6687.4315	0.0027	7	6	1	6	5	1
6991.4101	6991.4100	0.0001	8	5	3	7	4	3
6993.5040	6993.5085	-0.0045	8	5	4	7	4	4
7050.5187	7050.5204	-0.0017	7	7	1	6	6	1
7050.5187	7050.5204	-0.0017	7	7	0	6	6	0
7720.5779	7720.5694	0.0085	8	7	2	7	6	2
7720.5779	7720.5689	0.0090	8	7	1	7	6	1

Supplementary References

[1] Capriati, V., Florio, S., Luisi, R. & Salomone, A. Oxiranyl anion-mediated synthesis of highly enantiomerically enriched styrene oxide derivatives. *Organic Letters* **4**, 2445–2448 (2002).
